# Improving the N-terminal diversity of sansanmycin through mutasynthesis

**DOI:** 10.1186/s12934-016-0471-1

**Published:** 2016-05-06

**Authors:** Yuanyuan Shi, Zhibo Jiang, Xuan Lei, Ningning Zhang, Qiang Cai, Qinglian Li, Lifei Wang, Shuyi Si, Yunying Xie, Bin Hong

**Affiliations:** The Key Laboratory of Biotechnology of Antibiotics of Ministry of Health, Institute of Medicinal Biotechnology, Chinese Academy of Medical Sciences & Peking Union Medical College, No.1 Tiantan Xili, Beijing, 100050 China

**Keywords:** *m*-Tyr, *ssaX* deletion mutant, Mutasynthesis, Novel sansanmycin analogues

## Abstract

**Background:**

Sansanmycins are uridyl peptide antibiotics (UPAs), which are inhibitors of translocase I (MraY) and block the bacterial cell wall biosynthesis. They have good antibacterial activity against *Pseudomonas aeruginosa* and *Mycobacterium tuberculosis* strains. The biosynthetic gene cluster of sansanmycins has been characterized and the main biosynthetic pathway elucidated according to that of pacidamycins which were catalyzed by nonribosomal peptide synthetases (NRPSs). Sananmycin A is the major compound of *Streptomyces* sp. SS (wild type strain) and it bears a non-proteinogenic amino acid, *meta*-tyrosine (*m*-Tyr), at the N-terminus of tetrapeptide chain.

**Results:**

*ssaX* deletion mutant SS/XKO was constructed by the λ-RED mediated PCR targeting method and confirmed by PCR and southern blot. The disruption of *ssaX* completely abolished the production of sansanmycin A. Complementation in vivo and in vitro could both recover the production of sansanmycin A, and the overexpression of SsaX apparently increased the production of sansanmycin A by 20 %. Six new compounds were identified in the fermentation culture of *ssaX* deletion mutant. Some more novel sansanmycin analogues were obtained by mutasynthesis, and totally ten sansanmycin analogues, MX-1 to MX-10, were purified and identified by electrospray ionization mass spectrometry (ESI-MS) and nuclear magnetic resonance (NMR). The bioassay of these sansanmycin analogues showed that sansanmycin MX-1, MX-2, MX-4, MX-6 and MX-7 exhibited comparable potency to sansanmycin A against *M. tuberculosis* H_37_Rv, as well as multi-drug-resistant (MDR) and extensive-drug-resistant (XDR) strains. Moreover, sansanmycin MX-2 and MX-4 displayed much better stability than sansanmycin A.

**Conclusions:**

We demonstrated that SsaX is responsible for the biosynthesis of *m*-Tyr in vivo by gene deletion and complementation. About twenty novel sansanmycin analogues were obtained by mutasynthesis in *ssaX* deletion mutant SS/XKO and ten of them were purified and structurally identified. Among them, MX-2 and MX-4 showed promising anti-MDR and anti-XDR tuberculosis activity and greater stability than sansanmycin A. These results indicated that *ssaX* deletion mutant SS/XKO was a suitable host to expand the diversity of the N-terminus of UPAs, with potential to yield more novel compounds with improved activity and/or other properties.

**Electronic supplementary material:**

The online version of this article (doi:10.1186/s12934-016-0471-1) contains supplementary material, which is available to authorized users.

## Background

**Fig. 1:**
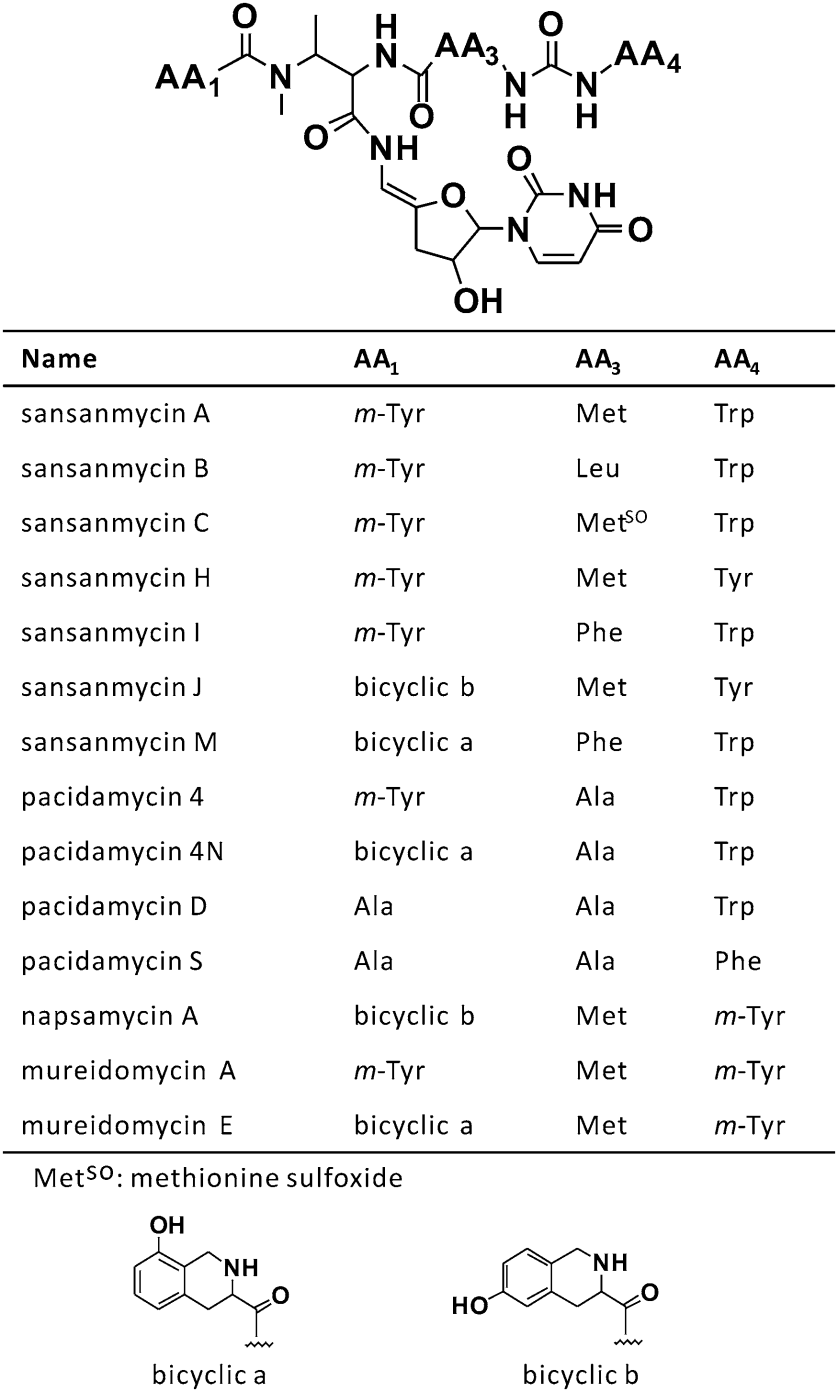
Structures of known uridyl peptide antibiotics

Sansanmycins [1], produced by *Streptomyces* sp. SS, belong to the uridyl peptide antibiotics (UPAs) including pacidamycins [2], napsamycins [3] and mureidomycins [4]. They hold a common and unique structure (Fig. 1), a 3′-deoxyuridyl attached to a pseudo-tetra/pentapeptidyl backbone via an exocyclic enamide. The peptidyl chain exhibited interesting double reversals due to the β-peptidation of the N-methyl-2,3-diaminobutyric acid (DABA) and a ureido linkage [5]. Sansanmycins exhibit good antibacterial activity against highly refractory pathogens including *Pseudomonas aeruginosa* and *Mycobacterium tuberculosis* [6]. With 1.5 million people killed by tuberculosis (TB) in 2014, the disease ranks alongside human immunodeficiency virus as a leading killer worldwide [7]. The increasing emergence of multi-drug-resistant (MDR) and extensive-drug-resistant (XDR) tuberculosis make the treatment more difficult. So there is an urgent need to develop novel anti-TB drugs with no cross-resistance to current clinically used antibiotics. Sansanmycins and other UPAs are of interest, due that they inhibit a clinically unexploited target MraY (phospho-MurNAc-pentapeptide translocase, also known as translocase I) [8], which catalyzes the transfer of UDPMurNAc-L-Ala-γ-D-Glu-*m*-DAP-D-Ala-D-Ala onto lipid carrier undecaprenyl phosphate to give lipid intermediate I in the bacterial peptidoglycan biosynthetic pathway. The potential bioactivity against *M. tuberculosis* coupled to its intriguing structure made this natural product a fascinating anti-TB lead compound.

Recently, the biosynthetic gene clusters for pacidamycins [5, 9], napsamycins [10], and sansanmycins [11] were identified and characterized, indicating that the assembly of the pseudo-tetrapeptide chain is catalyzed by nonribosomal peptide synthetases (NRPSs) with highly dissociated modules [12]. Besides, the biosynthesis of uridyl pentapeptide of pacidamycins was catalyzed by the tRNA-dependent aminoacyltransferase PacB, which transferred the alanyl residue from alanyl-tRNA to the N-terminus of the pseudo-tetrapeptide [13]. In contrast to ribosomal peptide synthesis, non-ribosomally assembled peptides contain not only the 20 proteinogenic amino acids but also many different building blocks, such as DABA, D-amino acids, hydroxyl amino acids, N- and C-methylated amino acids etc. Among them, non-proteinogenic amino acid *meta*-tyrosine (*m*-Tyr) is rarely found in bacterial secondary metabolites. UPAs and a potent cyclophilin inhibitor sanglifehrin A (SFA) are two examples containing *m*-Tyr as one of the building blocks. SfaA, identified in the SFA biosynthetic gene cluster, was speculated to catalyze the biosynthesis of *m*-Tyr [14]. As a homologue of SfaA in pacidamycin biosynthetic gene cluster, PacX was characterized as a phenylalanine 3-hydroxylase that catalyzed the synthesis of *m*-Tyr from L-phenylalanine (L-Phe) in vitro [15]. In sansanmycin biosynthetic gene cluster, SsaX is homologous to PacX with amino acid identity of 80 % across the whole protein, indicating that it is responsible for the biosynthesis of *m*-Tyr in *Streptomyces* sp. SS.

Although natural UPAs have potential to treat refractory infections, there is no UPAs entering clinical trials until now mainly due to their relatively poor physicochemical property. In previous studies, the N-terminal amino acid of the tetrapeptide of UPAs was supposed to be important functional group for the inhibition of MraY [16, 17]. It was proposed that the protonated ammonium ion binds in place of the Mg^2+^ cofactor at the MraY active site via *cis*-amide linkage [17]. The N-terminal amino acid of known UPAs is almost dominated by *m*-Tyr or different bicyclic acids (possibly derived from *m*-Tyr), except that some pacidamycins possess Ala instead (Fig. 1). In this study, we focus on the substitution of the N-terminal amino acid to get novel sansanmycin analogues by mutasynthesis. Mutasynthesis is a useful method in the generation of new antibiotic derivatives [18]. This approach could expand the chemical diversity of secondary metabolites and produce novel compounds with improved physicochemical properties or altered bioactivity. For example, it has been successfully employed to get novel nucleoside antibiotics such as nikkomycin analogues [19] and new ansamycin derivatives [20]. However, mutational biosynthesis has not been employed to obtain UPA derivatives so far.

Here, we demonstrated that SsaX is responsible for the biosynthesis of *m*-Tyr in vivo by gene deletion and complementation and the sansanmycin production could be increased through the overexpression of *ssaX*. Six new sansanmycin analogues were purified and characterized in *ssaX* deletion mutant, indicating the substrate flexibility of the responsible NRPS. To expand the diversity of sansanmycins by mutasynthesis, different types of substrates were fed to the *ssaX* deletion mutant and some novel sansanmycin derivatives were obtained. These compounds were purified and structurally identified, some of which exhibited improved antibacterial activity or stability.

## Results

### In-frame deletion of *ssaX* and its complementation

**Fig. 2:**
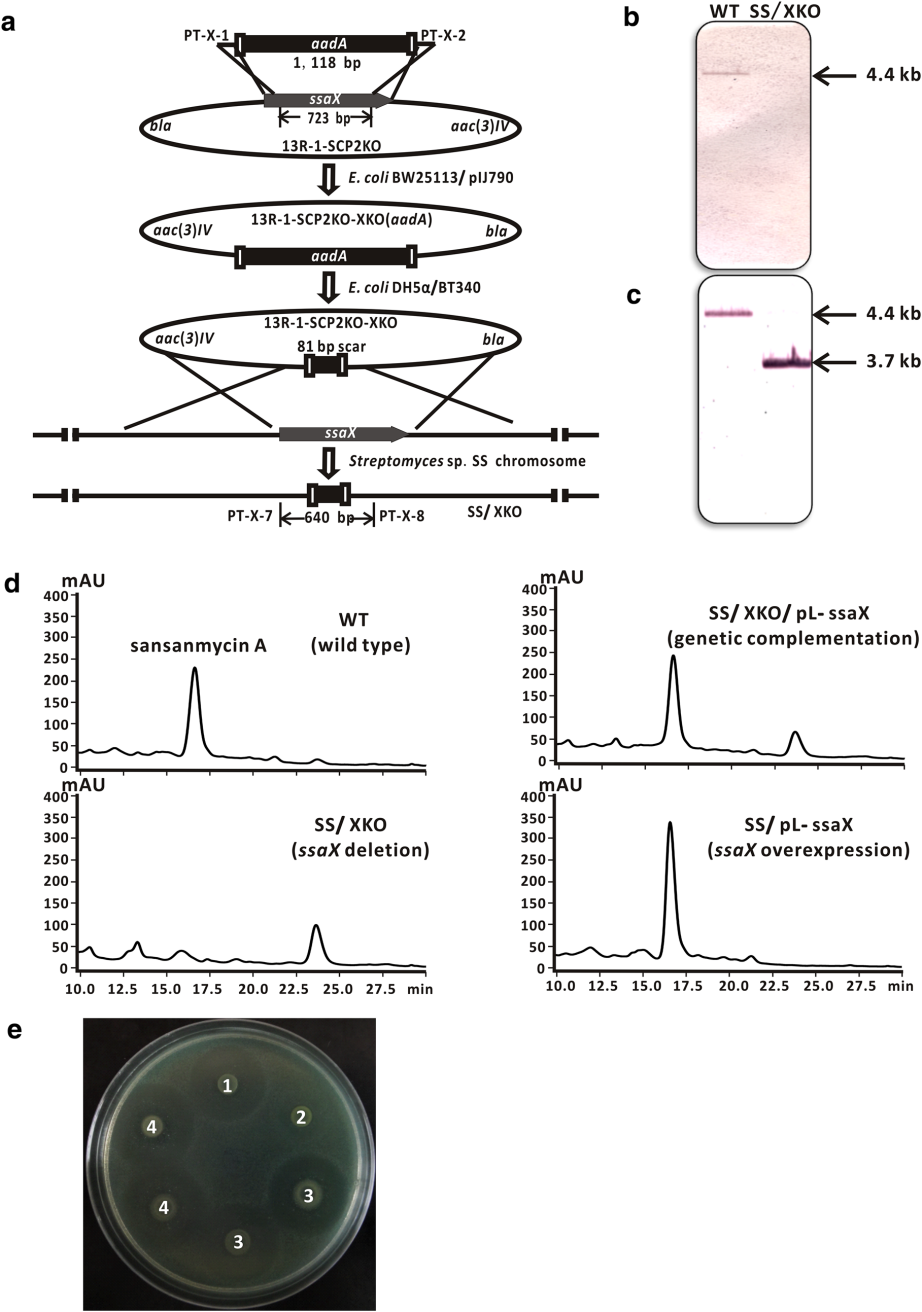
Effects of the deletion of *ssaX* on sansanmycin production. **a** Construction of the *ssaX* deletion mutant (SS/XKO) by PCR targeting and its PCR verification (using primers PT-X-7 and PT-X-8). Primers PT-X-1 and PT-X-2 were used to amplify a streptomycin resistance cassette (*aadA* gene), which substituted *ssaX* on cosmid 13R-1-SCP2KO. *bla*, ampicillin resistance marker; *aac(3)IV*, apramycin resistance marker. **b**, **c** Southern blot hybridization of wild type strain and SS/XKO. The genomic DNAs were digested with *Bam*HI and hybridized with specific probes of *ssaX* deleted fragment (**b**) and the fragment downstream of *ssaX* (**c**) respectively. The wild type strain showed the hybridized band of 4.4 kb both in **b** and **c**. The correct *ssaX* deletion mutant showed no hybridized band in **b** and the hybridized band of 3.7 kb instead of 4.4 kb in **c**. **d** HPLC analysis of different strains. Sansanmycin A is the major compound in wild type strain with *m*-Tyr at N-terminus. **e** Antibacterial activity analysis of different strains. 1, wild type strain; 2, SS/XKO; 3, SS/XKO/pL-ssaX; 4, SS/pL-ssaX

In order to investigate the contribution of *ssaX* to sansanmycin biosynthesis, an *ssaX* deletion mutant SS/XKO was constructed from *Streptomyces* sp. SS by PCR targeting [21] using cosmid 13R-1 [11] which contains *ssaX* and the majority of other biosynthetic genes. Cosmid 13R-1-SCP2KO was firstly constructed from cosmid 13R-1 with the minimal replicon of SCP2* replaced by ampicillin resistance gene in order to promote homologous recombination for the disruption of *ssaX*. Then the *ssaX* gene in 13R-1-SCP2KO was in-frame deleted and the resulted 13R-1-SCP2KO-XKO was introduced into the wild type strain by conjugation (Fig. 2a). The *ssaX* in-frame deletion mutant SS/XKO was verified by PCR using primers PT-X-7 and PT-X-8 (Fig. 2a) and further confirmed by southern blot analysis (Fig. 2b, c). The coding region of *ssaX* was cloned into a pSET152 [22] -derived expression plasmid, pL646 [23], under the control of a strong constitutive promoter, ermE*p, to give pL-ssaX. The plasmid was introduced into SS/XKO strain by conjugation and the complementary strain SS/XKO/pL-ssaX was resulted to exclude the potential polar effect in SS/XKO. Metabolites of constructed strains were scrutinized to compare with that of the wild type strain by HPLC, LC/MS and bioassay analysis. The major product in the wild type strain, sansanmycin A with *m*-Tyr at the N-terminus of tetrapeptide chain, was not detected in SS/XKO (Fig. 2d). The culture broth of SS/XKO showed no antibacterial activity against *P. aeruginosa* 11, which was used as the test microorganism for the bioassay of sansanmycins (Fig. 2e). Meanwhile, the complementary strain SS/XKO/pL-ssaX completely recovered the production of sansanmycin A detected by HPLC analysis (Fig. 2d) and antibacterial bioassay (Fig. 2e).

**Fig. 3:**
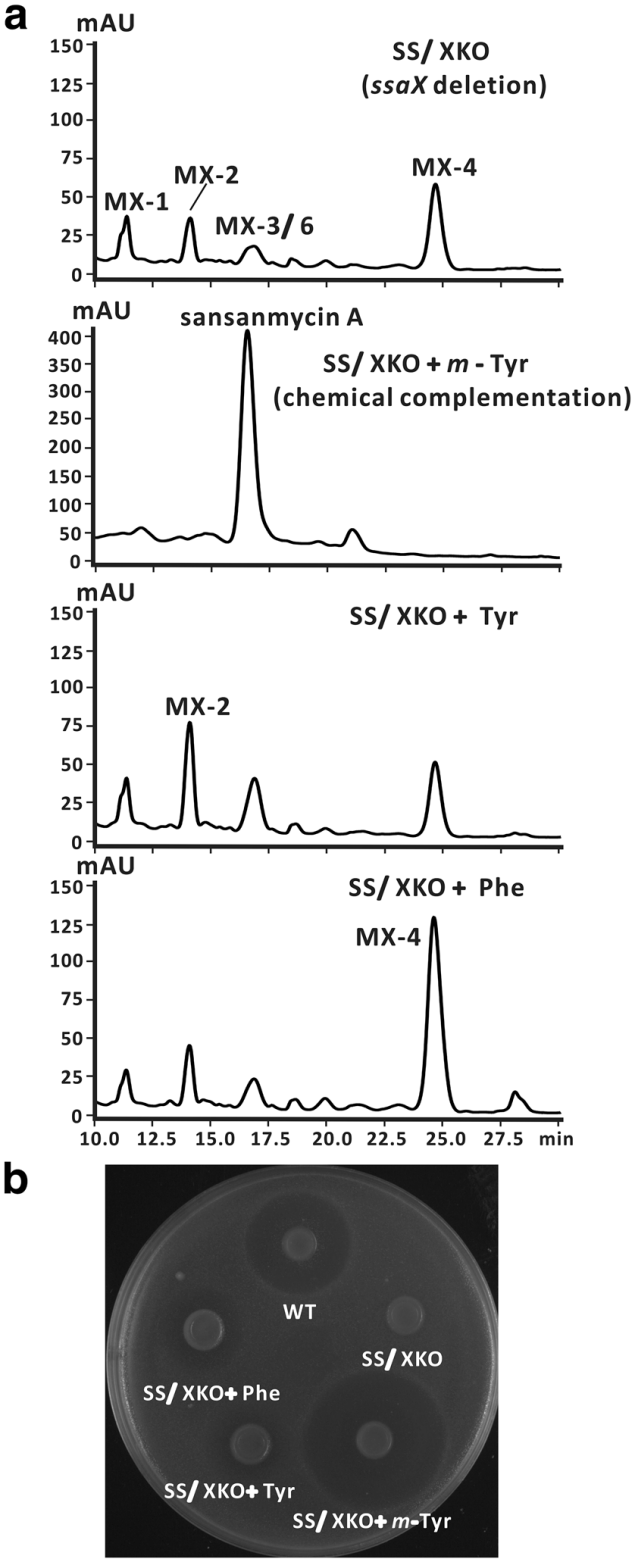
Effects of supplying SS/XKO with *meta*-tyrosine (*m*-Tyr), tyrosine (Tyr) and phenylalanine (Phe). **a** HPLC analysis of the culture broth of different supplementations. The final concentration of each exogenous substrate was 3 mM. **b** Antibacterial activity analysis of the culture broth of different supplementations

In addition to genetic complementation in vivo, a chemical complementation by feeding the substrate *m*-Tyr (3 mM) to the fermentation medium of the *ssaX* deletion mutant SS/XKO was also performed. The result of HPLC showed that the major product of this culture broth was sansanmycin A and the production level was significantly higher than the wild type strain (Fig. 3a). Consistent with this result, the inhibition zone was bigger than that of wild type strain in the bioassay (Fig. 3b). All of these results demonstrated that SsaX was responsible for the biosynthesis of *m*-Tyr in sansanmycin biosynthetic pathway.

To further investigate the role of *ssaX* in sansanmycin biosynthesis, the plasmid pL-ssaX was transferred into the wild type strain by conjugation to give the *ssaX* overexpression strain SS/pL-ssaX, with pSET152 transferred strain SS/pSET152 as a control. With the same growth curves, the overexpression of SsaX apparently increased the production of sansanmycin A by 20 % from HPLC analysis (Fig. 2d). The antibacterial activity against *P. aeruginosa* 11 also showed that SS/pL-ssaX exhibited bigger inhibition zone than the control strain (Fig. 2e). This result, together with the result of chemical complementation, suggested that the biosynthesis of *m*-Tyr catalyzed by SsaX is at least one of the rate-limiting steps in the sansanmycin production [24].

### Isolation and structural determination of sansanmycin analogues in *ssaX* deletion mutant

Through the HPLC and LC/MS analysis, a series of minor components of sansanmycin analogues were detected in the cultivated broth of *ssaX* deletion mutant SS/XKO. In order to characterize these compounds, SS/XKO fermentations were scaled up to obtain enough amount of material for further analysis. The target compounds were enriched by macroporous absorbant resin from fermentation broth, then isolated using DEAE-Sephadex A25 guided by HPLC–UV to yield the crude sansanmycin analogues. Subsequently, the crude compounds were purified by preparative HPLC. As a result, six new sansanmycin analogues were obtained and designated as sansanmycin MX-1 to MX-6 respectively. Their structures were elucidated by electrospray ionization mass spectrometry (ESI-MS), ESI-MS/MS and nuclear magnetic resonance (NMR) spectroscopic analysis.

**Fig. 4:**
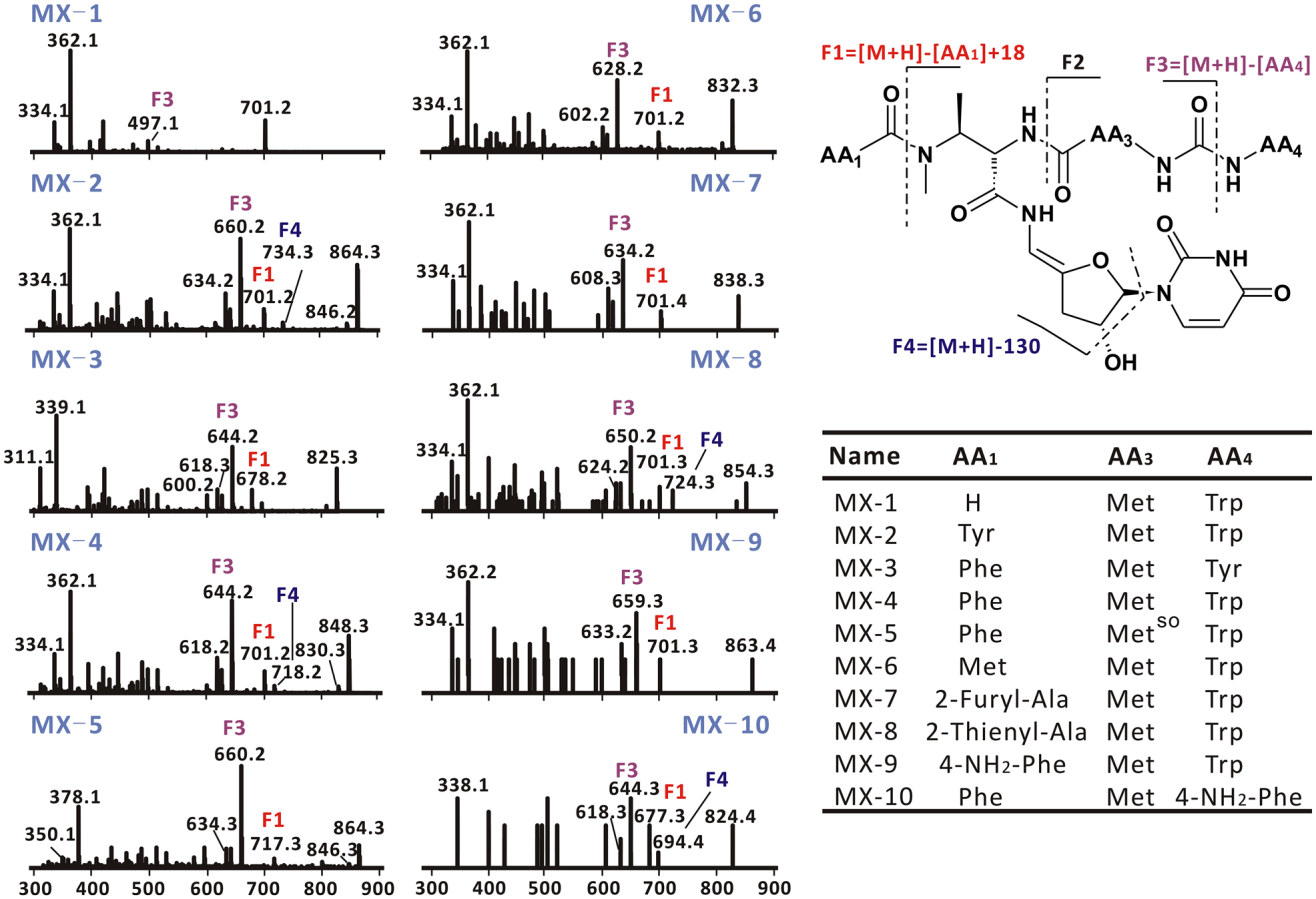
(+)-ESI-MS/MS data and structures of ten new sansanmycin analogues

**Fig. 5:**
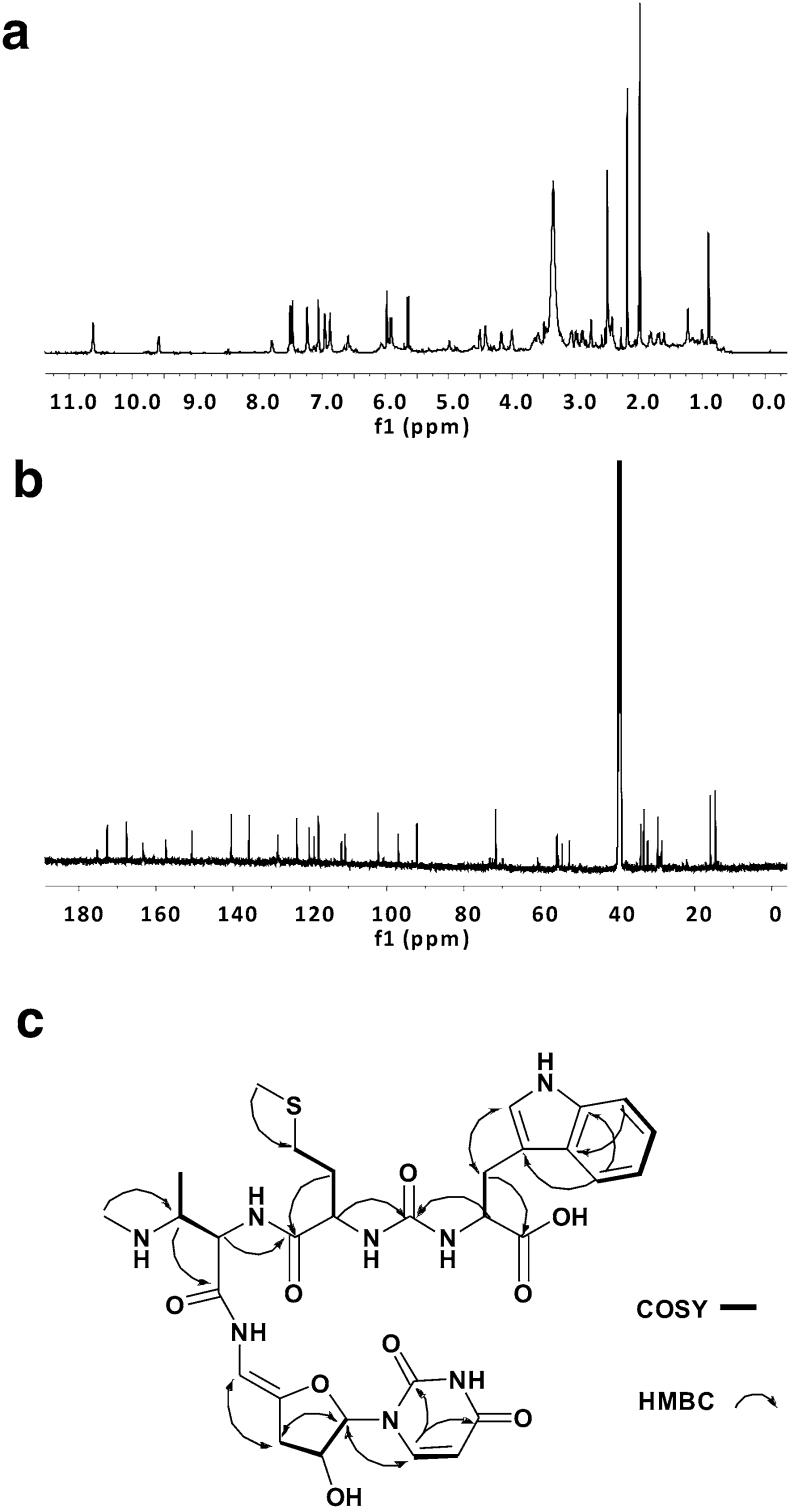
Structural determination of sansanmycin MX-1. **a** ^1^H NMR spectrum of sansanmycin MX-1 (600 MHz, DMSO-*d*_*6*_) **b** ^13^C NMR spectrum of sansanmycin MX-1 (150 MHz, DMSO-*d*_*6*_) **c** Selected 2D NMR correlations for sansanmycin MX-1

Sansamycin MX-1, MX-2, MX-4 and MX-6 have molecular weights of 700, 863, 847 and 831, respectively. The ESI-MS/MS results of them (Fig. 4) showed the same fragment patterns with sansanmycin A. All of these four compounds showed the same diagnostic fragment with sansanmycin A, m/z 701, corresponding to the loss of the N-terminal amino acid (AA_1_), suggesting they varied at the N-terminal amino acid. In support of this hypothesis, the ^1^H NMR data for MX-1 (Fig. 5a; Additional file 1: Table S1), MX-2 (Additional file 1: Table S2, Figure S4), MX-4 (Additional file 1: Table S3, Figure S6) and MX-6 (Additional file 1: Table S4, Figure S8) proved to be very similar to that of sansanmycin A, with differences limited to replacement of the N-terminal *m*-Tyr in sansanmycin A [δ 7.23 (t, 1H), 6.78 (d, 1H), 6.75 (d, 1H), 6.72 (s, 1H), 4.04 (m, 1H), 2.51 (m, 1H), 2.89 (m, 1H)] [1] with a hydrogen in MX-1 (δ 10.78), a tyrosine (Tyr) in MX-2 [δ 7.51 (d, 2H), 6.97 (d, 2H), 4.15 (m, 1H), 3.08 (m, 1H), 2.97 (dd, 1H)], a Phe in MX-4 [δ 7.34 (m, 2H) 7.15 (m, 2H), 7.27 (m, 1H), 3.92 (m, 1H), 2.73 (m, 1H), 2.86 (m, 1H)] and a methionine (Met) in MX-6 [δ 4.31 (m, 1H), 2.47 (m, 1H), 2.37 (m, 1H), 1.99 (s, 3H), 1.81 (m, 1H)]. Interpretation of the ^13^C (Fig. 5b; Additional file 1: Figure S5, S7, S9) and 2D NMR data (Fig. 5c; Additional file 1: Figures S1–S3) also confirmed these proposed structures.

MX-3 has a molecular weight of 824, 16 mass units smaller than that of sansanmycin H [25], attributed to the loss of an oxygen atom. Furthermore, the ESI-MS/MS spectrum of MX-3 also showed the same diagnostic fragment with sansanmycin H, m/z 678 for the loss of the N-terminal *m*-Tyr, which suggested that the *m*-Tyr in sansanmycin H was replaced by a Phe in MX-3. Comparison with that of sansanmycin H, the ^1^H NMR spectrum of MX-3 (Additional file 1: Figure S10) showed a different aromatic pattern from sansanmycin H, with a Phe [δ 7.15 (m, 2H), 7.15 (m, 2H), 7.27 (m, 1H)] instead of *m*-Tyr [δ 7.23 (t, 1H), 6.78 (d, 1H), 6.75 (d, 1H), 6.72 (s, 1H)] [25].

MX-5 has a molecular weight of 863, 16 mass units greater than MX-4, corresponding to an extra oxygen atom. Comparison with that of MX-4, the ^1^H NMR spectrum of MX-5 (Additional file 1: Figure S11) showed a downfield shifted methyl proton signal [from δ 2.01 (−SCH_3_) to 2.46 (−SOCH_3_)], which hinted the oxidation of MX-4 to MX-5. The ESI-MS/MS analysis (Fig. 4) further confirmed this hypothesis.

The obtained six compounds MX-1–6 were new members of sansanmycin family. Compared with SS-A, MX-1, short of the N-terminal *m*-Tyr, bears a tri-pseudopeptide backbone that was found in the family of UPAs for the first time. It is the accumulated precursor when there is no *m*-Tyr present in SS/XKO, which is also the obvious evidence that SsaX catalyzes the biosynthesis of *m*-Tyr. The other five compounds were different from the known sansanmycins by the virtue of bearing Tyr, Phe and Met at the N-terminus, which were firstly reported in the family of UPAs. The presence of the new sansanmycin analogues with various N-terminal amino acids hinted that the NRPS responsible for the incorporation of the N-terminal amino acid into the tetra-pseudopeptide backbone has moderate substrate promiscuity, suggesting that certain amounts of sansanmycin analogues might be able to be generated by mutational biosynthesis using *ssaX* deletion mutant SS/XKO.

### Generation of structurally diverse sansanmycin analogues using *ssaX* deletion mutant

Initially twenty proteinogenic amino acids including Phe, Tyr and Met were used to probe the feasibility of mutasynthesis. The production of sansanmycin MX-2 which bears Tyr as its N-terminus was nearly doubled when fed SS/XKO with Tyr (3 mM) (Fig. 3). Similarly, the production of sansanmycin MX-4 which bears Phe as its N-terminus was increased to two to three times when fed with Phe (3 mM) (Fig. 3). But the HPLC profile of the fermentation broth of *ssaX* deletion mutant fed with Met, as well as other proteinogenic amino acids (3 mM) had no obvious changes (data not shown). This may be explained by the substrate preference of the NRPS, which preferred to select Phe or Tyr rather than any other proteinogenic amino acids. This result is consistent with the production level of sansanmycin analogues in SS/XKO. The improved production of sansanmycin MX-2 and MX-4 by feeding substrates Phe and Tyr suggested that mutasynthesis might be suitable to produce sansanmycin analogues with alternate N-terminal substrate.

**Fig. 6:**
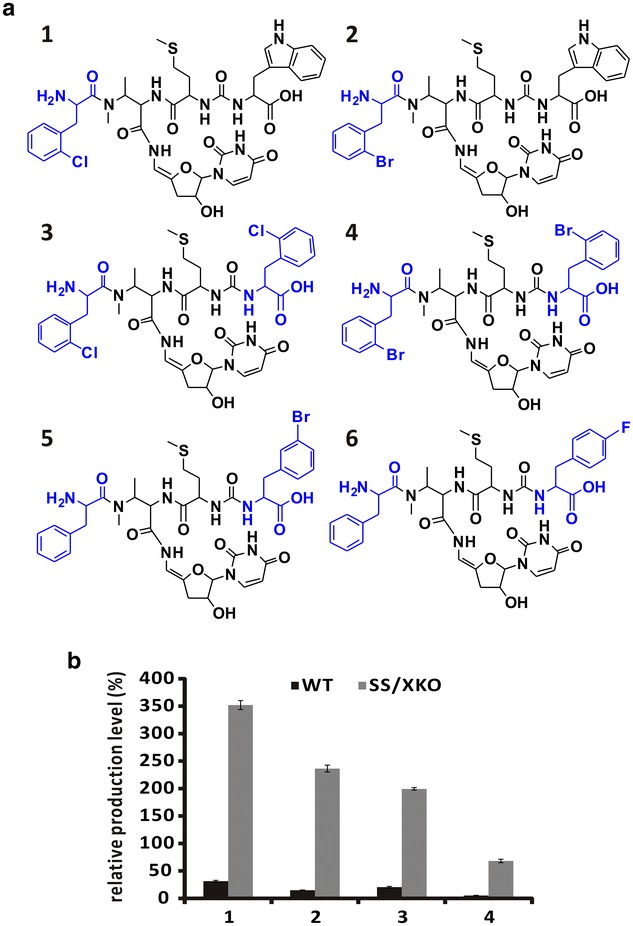
The production of novel sansanmycin analogues. **a** The structures of novel compounds produced by *ssaX* deletion mutant SS/XKO fed with halogenated Phe. The final concentration of each exogenous substrate was 3 mM. The *blue* labeled parts represent the halogenated Phe incorporated into N- and/or C-terminus. **b** The relative production level of novel sansanmycin derivatives 1–4 in different strains. Chloramphenicol was added to the cultures as an extra standard

Incorporation of halogens into the molecules might exert a significant effect on their physicochemical properties of the products [26, 27]. Halogenated Phe had been fed to get sansanmycin analogues in the wild type strain by Xie et al. and they were preferably incorporated at the C-terminus (AA_4_) [28]. In order to incorporate halogenated Phe into sansanmycin analogues at the N-terminus efficiently, the *ssaX* deletion mutant SS/XKO was evaluated. Some new sansanmycin analogues were produced by SS/XKO and their structures were determined by ESI-MS/MS. These sansanmycin analogues may be divided into three main groups (Fig. 6a). Compounds in the first and second groups were with halogenated Phe incorporated into the N-terminus (e.g., compounds 1 and 2) or both the N-terminus and the C-terminus (e.g., compounds 3 and 4) respectively. The third group was with halogenated Phe incorporated into the C-terminus and with Phe at the N-terminus instead of *m*-Tyr (e.g., compounds 5 and 6). What’s more, the production level of the analogues with halogenated Phe at the N-terminus was increased remarkably compared with the wild type strain with the same growth curves. For example, when fed with 2-chloro- and 2-bromophenylalanines (3 mM), the production level of compound 1 and 2 was 11- and 16-fold higher in SS/XKO respectively compared with the wild type strain (Fig. 6b). Similarly, compound 3 and 4 were increased 10- and 13-fold respectively (Fig. 6b). In a word, the *ssaX* deletion mutant was a better host to get sansanmycin analogues with N-terminus substitution, for its absence of the endogenous optimal substrate *m*-Tyr.

**Fig. 7:**
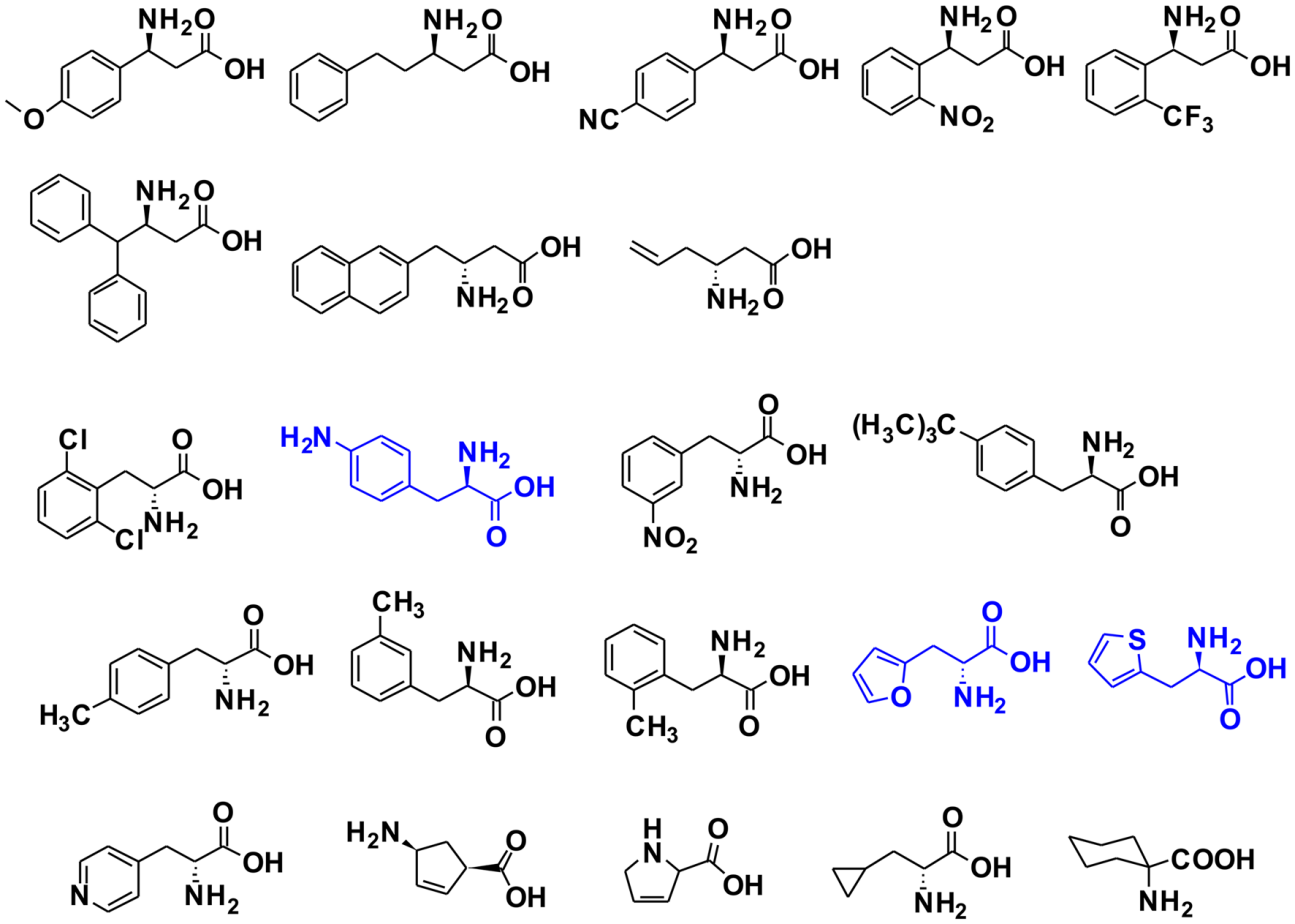
Structures of the commercially available non-proteinogenic amino acids fed to SS/XKO. *Blue* labeled substrates represent the non-proteinogenic amino acids successfully incorporated into the N-terminus. The final concentration of each exogenous substrate was 3 mM

Motivated by the results of above feeding experiments and in order to expand the diversity of sansanmycins, more than 20 commercially available non-proteinogenic amino acid analogues were fed to SS/XKO (3 mM), including α-amino acids and β-amino acids with diverse side chains, such as aliphatic groups, substituted aromatic phenyl groups, as well as aromatic and nonaromatic heterocyclic groups (Fig. 7). The results showed that β-amino acids detected here couldn’t be incorporated into the peptidyl chain of sansanmycins. When feeding methyl substituted phenylalanines, the major products were the C-terminal derivatives with Phe at the N-terminus. When supplemented with *para*-amino-phenylalanine, two products with the fed precursor incorporated at the N- (MX-9) and the C-terminus (MX-10) respectively were obtained. Unexpectedly, two aromatic heterocyclic α-amino acids, 2-furylalanine and 2-thienylalanine, can be incorporated into the N-terminus to produce corresponding derivatives MX-7 and MX-8, respectively. The structures of MX-7–10 were determined by ESI-MS/MS (Fig. 4). MX-7, MX-8 and MX-9 showed the expected molecular weights and the same diagnostic fragment with sansanmycin A, m/z 701 for the loss of the N-terminal amino acid, suggesting the precursors added were incorporated into the N-terminus (Fig. 4). MX-10 displayed the same fragment with MX-4, m/z 644 corresponding to the loss of the C-terminal amino acid, which suggested the *p*-amino-phenylalanine administrated was incorporated into the C-terminus (Fig. 4).

The yield of each sansanmycin derivative produced by mutasynthesis was estimated by the peak areas from HPLC (Table 1). The production level of sansanmycin MX-4 in SS/XKO without supplementation was considered as 100 %. When fed with *m*-Tyr, production of sansanmycin A was the highest. Surprisingly, the production level of MX-7 bearing 2-furylalanine was much higher than that of MX-2 bearing Tyr. All of these results suggested that other unnatural substrates might be acceptable for the mutant strain, especially aromatic α-amino acids.

**Table 1:** Comparison of the relatively production level of sansanmycin analogues in *ssaX* deletion mutant SS/XKO

Fed substrate (3 mM)	Sansanmycin analogue	Production level (%)
─	sansanmycin MX-4	100
*m*-Tyr	sansanmycin A	~350
Phe	sansanmycin MX-4	~280
2-Cl/Br-Phe	compound 1, 2	240–270
2-Furyl-Ala	sansanmycin MX-7	~240
Tyr	sansanmycin MX-2	~80
2-Thienyl-Ala	sansanmycin MX-8	~35
4-NH_2_-Phe	sansanmycin MX-9	~30

### Antibacterial activity and stability of novel sansanmycin analogues

The obtained sansanmycin analogues MX-1–10 were tested for their antibacterial activity against different bacteria including gram-negative bacteria, gram-positive bacteria as well as *M. tuberculosis* H_37_Rv and clinically isolated MDR and XDR *M. tuberculosis* strains (Table 2). As expected, all the tested compounds (except MX-3) displayed different degrees of activity against *E. coli ΔtolC* mutant strain, which was consistent with previous results that UPAs can be exported by the AcrAB-TolC efflux system in *E. coli* [29]. Among them, sansanmycin MX-2 and MX-6 remained potency against *P. aeruginosa* equivalent to sansanmycin A. Interestingly, compound MX-6 exhibited antibacterial activity against gram-positive *B. subtilis*, which was not found in natural UPAs. Sansanmycin MX-1, MX-2, MX-4, MX-6 and MX-7 showed potency against *M. tuberculosis* H_37_Rv comparable to sansanmycin A. More noticeably, these tested compounds exhibited equivalent potency against clinically isolated MDR and XDR strains. These results maybe lie in the fact that the target of UPAs is clinically unexploited.

**Table 2:** Activities of sansanmycin analogues

Compounds (sansanmycin)	MIC (μg/ml)						
	*E. coli ΔtolC*	*P. aeruginosa* 11	*B. subtilis* CMCC (B) 63501	*M. tuberculosis*			
				H_37_Rv	FJ05189	FJ05120	FJ05195
MX-1	32	>128	>128	16	8	8	8
MX-2	2	32	>128	16	16	8	8
MX-3	>128	>128	64				
MX-4	8	64	>128	8	16	8	16
MX-5	8	>128	>128				
MX-6	4	32	16	8	8	8	8
MX-7	8	128	>128	16	8	8	16
MX-8	8	128	>128				
MX-9	16	128	>128				
MX-10	64	>128	>128				
A	2	16	>128	16	16	16	8
Streptomycin	16	4	2	0.25	<0.06	<0.06	>128
Isoniazid				0.125	8	16	>128
Rifampicin				<0.06	4	>128	>128
Ethambutol				0.5	2	0.5	>128

*M. tuberculosis* H_37_Rv, standard and susceptible strain. FJ05189, FJ05120 and FJ05195 are clinical isolates of *M. tuberculosis*. FJ05189 and FJ05120, MDR strains, resistant to isoniazid and rifampicin; FJ05195, XDR strain, resistant to isoniazid, rifampicin, ethambutol, streptomycin, kanamycin and ofloxacin

**Fig. 8:**
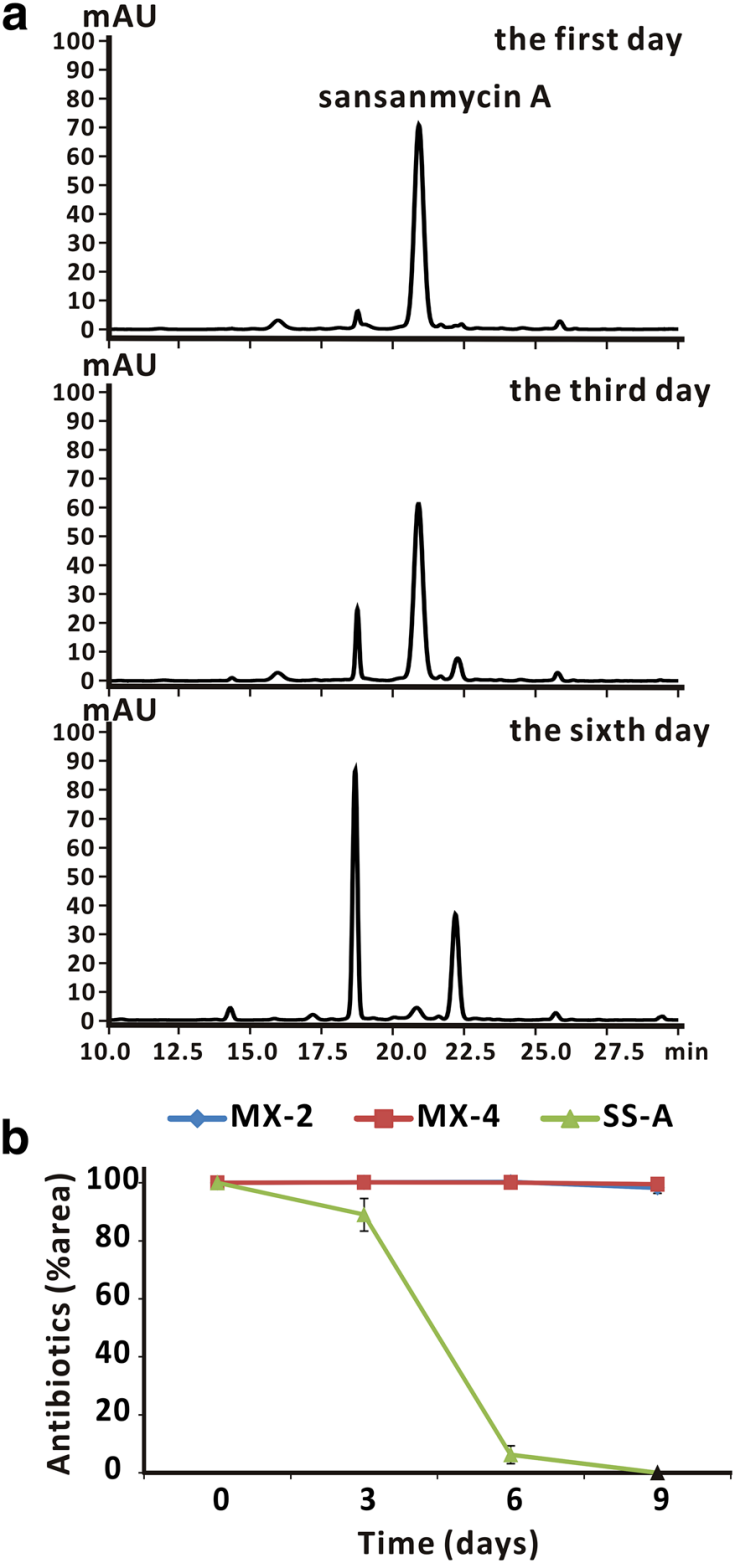
Stability of sansanmycin A, MX-2 and MX-4. **a** HPLC analysis of sansanmycin A at indicated days in KH_2_PO_4_ buffer (pH 6.0) at room temperature. **b** The changes of the level of sansanmycin A, MX-2 and MX-4 over time. All samples were analyzed by HPLC and quantified according to the areas of peaks. *blue line*, sansanmycin MX-2; *red line*, sansanmycin MX-4; *green line*, sansanmycin A

During the early stage of drug development of sansanmycin A, we found that the structure of sansanmycin A was not stable at room temperature (Fig. 8a). As it is easier to get enough amount of sansanmycin MX-2 and MX-4, the stability test of sansanmycin A, MX-2 and MX-4 was performed in KH_2_PO_4_ buffer (pH 6.0) at room temperature. Under this condition, sansanmycin MX-2 and MX-4 kept mostly unchanged after 6 days of incubation whereas less than 10 % sansanmycin A was remained (Fig. 8b), which suggested that sansanmycin MX-2 and MX-4 are more stable than sansanmycin A.

## Discussion

During the past decade, considerable efforts have been made to exploit new UPA derivatives. Seventeen sansanmycin analogues were semi-synthesized with sansanmycin A as the starting material, but most of them exhibited less anti-mycobacterial activity in comparison with parent natural product [30]. Strategy of precursor-directed biosynthesis was employed to get pacidamycin analogues with modified C-terminal amino acid through feeding Trp derivatives [26]. The same strategy was also applied to sansanmycin-producing strain, resulting sansanmycin analogues with the C-terminus substituted by Phe derivatives [28]. Although some UPA analogues were produced, few of them had significantly improved antibacterial activity and/or physicochemical property. In the past 5 years, the biosynthetic pathways have been studied extensively at the genetic, enzymatic and regulatory levels [9–13], and bioengineering approaches are available to be used in producing novel UPA derivatives. In this work, mutational biosynthesis is employed by blocking the biosynthesis of *m*-Tyr and then feeding variety of alternative substrates to produce novel sansanmycin derivatives. This strategy is efficient to obtain novel sansanmycin analogues, creating a great structural diversity at the N-terminus.

In most of the reported UPAs, the N-terminus of the tetra-pseudopeptide (AA_1_) was occupied by *m*-Tyr and its related bicyclic acids, except that pacidamycin D and S have an Ala at the N-terminus (Fig. 1). Two NRPSs responsible for selection of amino acid to incorporate into the N-terminus of pacidamycin have been reported. PacU was demonstrated to specifically activate Ala [5], and PacW was identified to activate *m*-Tyr [12]. In sansanmycin-producing strain, there are also two homologues of PacU, SsaU and SsaW, existed in sansanmycin biosynthetic gene cluster [11], but their amino acid sequences were almost exactly the same, with only one alteration of Lys to Arg [11]. Though they exhibited high overall homology with both PacU and PacW, the main residues in the binding pocket (specificity-conferring code) in the adenylation (A) domain [31, 32] were same with PacW but different from PacU. This is consistent with the fact that *m*-Tyr is the optimum substrate for the production of sansanmycin A in the wild type strain, and all the natural sansanmycin derivatives have *m*-Tyr and its related bicyclic acids at the N-terminus. When the biosynthesis of *m*-Tyr is blocked, Phe, Tyr, and small amount of Met may incorporate into the polypeptide of sansanmycin in *ssaX* deletion mutant, showing the potential of this mutant as a cell factory to expand the chemical diversity of AA_1_.

Various substrates including Phe, Tyr, halogenated Phe and other non-proteinogenic amino acids were fed to the *ssaX* deletion mutant and about 20 novel sansanmycin analogues with different N-termini were produced according to the substrate promiscuity of the A domain of SsaU and SsaW. Ten of these compounds were purified and structurally determined by ESI-MS, ESI-MS/MS (Fig. 4) and NMR. According to the production level of the compounds in *ssaX* deletion mutant fed with corresponding substrate (Table 1), the preference of the A domain in charge of activating the sansanmycin N-terminal amino acid is *m*-Tyr > Phe ≥ *ortho*-halogenated phenylalanines ≥ 2-furylalanine > Tyr > 2-thienylalanine ≥ *p*-amino-phenylalanine > Met. Out of our expectation, 2-furylalanine and 2-thienylalanine could be incorporated into the sansanmycin, suggesting the diversity of AA_1_ might be worth to explore further by trying more structurally diverse substrates. The compounds produced by mutasynthesis in this study retained the anti-TB activity of sansanmycin A in vitro, and more encouragingly, they showed similar activity to MDR and even XDR strains isolated from patients. Meanwhile, the stability of MX-2 and MX-4 was demonstrated to be greatly improved compared to sansanmycin A.

From the result of previous researches on the UPA derivatives of the wild type strains [8, 25] or by precursor-directed biosynthesis [26, 28], the A domain responsible for activating the C-terminal amino acid (AA_4_) has relatively high substrate promiscuity from Trp, Tyr to Phe and substituted Phe and Trp. On the other hand, the amino acid at the position of AA_3_ also can vary from Met and Ala [8] to Leu and Phe in sansanmycins [25, 33]. Now, together with the diversity at AA_1_ produced in this study, the combination of the variations in these three parts of the polypeptide backbone could be potentially expected that hundreds of new sansanmycin analogues might be obtained. Recently, crystal structure of *Aquifex aeolicus* MraY has been published [34], and the residues (Asp^117^, Asp^118^, Asp^265^, and His^324^) important for the activity of MraY in the active site have been elucidated [34]. The structural information of MraY from *A. aeolicus* sets foundations for homologous modeling of MraY from *M. tuberculosis* [34, 35], which will facilitate the study on structure activity relationship (SAR) of novel chemically diverse UPA derivatives obtained by further rationale genetic engineering manipulation.

## Conclusion

It is demonstrated that SsaX is responsible for the biosynthesis of *m*-Tyr in vivo by gene deletion and complementation and the sansanmycin production could be increased through the overexpression of *ssaX*. Six new sansanmycin analogues were purified and characterized in *ssaX* deletion mutant, indicating the substrate flexibility of the responsible NRPS. The diversity of sansanmycin was further expanded by mutasynthesis, in which different types of substrates were fed to the culture of *ssaX* deletion mutant. Totally ten compounds were purified, structurally identified and firstly reported. Five of them displayed anti-mycobacterial activity comparable to sansanmycin A and especially, they are active to MDR and even XDR *M. tuberculosis* clinical strains. In addition, sansanmycin MX-2 and MX-4 displayed significantly improved stability than sansanmycin A. These improved properties may promote the novel anti-TB drug investigation targeting a clinically unexploited target MraY.

## Methods

### Strains, plasmids and growth conditions

The wild-type *Streptomyces* sp. SS strain, obtained from China Pharmaceutical Culture Collection (CPCC 200442), was used as a host strain for the propagation and disruption of genes, as described previously [11]. *Streptomyces* sp. SS and its derivatives were grown at 28 °C on solid S5 medium [36] for sporulation and in the liquid fermentation medium [1] for the production of sansanmycins. Mannitol soya flour (MS) agar [37] and liquid phage medium [38] were used for conjugation and isolation of genomic DNA respectively. *Escherichia coli* DH5α was used as a host for general cloning experiments [39]. *E*. *coli* ET12567/pUZ8002 [40] was used for conjugal transfer according to the established protocol [37]. *E. coli* BW25113/pIJ790 was used as the host for Red/ET-mediated recombination [21]. *E. coli* DH5α containing the temperature sensitive FLP recombination plasmid BT340 was used as the host to remove the central part of the disruption cassette [21]. *E. coli* DH5α and ET12567/pUZ8002 were incubated in Luria–Bertani medium (LB) [39] at 37 °C. *E. coli* BW25113/pIJ790 and DH5α/BT340 were grown at 30 °C in LB medium. *P. aeruginosa* 11, the indicator strain for the antibacterial bioassay of culture broth of different *Streptomyces* sp. SS strains [6], was grown on F403 agar [36]. *E. coli ΔtolC* mutant, *P. aeruginosa* 11, *Bacillus subtilis* CMCC (B) 63501, *M. tuberculosis* H_37_Rv and clinically isolated strains were used for testing antimicrobial activity for the compounds. When required, strains were incubated with apramycin (Am, 50 μg/ml), ampicillin (Amp, 100 μg/ml), spectinomycin (Spec, 50 μg/ml), kanamycin (Km, 50 μg/ml) and chloramphenicol (Cm, 30 μg/ml). All the strains and plasmids used in this study are listed in Table 3.

**Table 3:** Strains and plasmids used in this study

Strains/plasmids	Relevant characteristics	Reference
*Strains*		
*Streptomyces* sp. SS	Wild-type strain (sansanmycin-producing strain), CPCC 200442	[1]
SS/XKO	Mutant of *Streptomyces* sp. SS with the in-frame deletion of *ssaX*	This study
SS/XKO/pL-ssaX	SS/XKO with the expression vector pL-ssaX, Am^r^	This study
SS/pL-ssaX	*Streptomyces* sp. SS with the expression vector pL-ssaX, Am^r^	This study
*Escherichia coli*		
DH5α	General cloning host	[39]
ET12567/pUZ8002	Donor strain for intergeneric conjugation between *E. coli* and *Streptomyces*, Cm^r^, Km^r^	[40]
BW25113/pIJ790	Strain for RED/ET-mediated recombination, Cm^r^	[21]
*ΔtolC* mutant	Strain for testing antimicrobial activity	[44]
*Pseudomonas* *aeruginosa* 11	Strain for sansanmycin bioassays	[6]
*Bacillus subtilis* CMCC (B) 63501	Strain for testing antimicrobial activity	
*Mycobacterium tuberculosis*	Strain for testing antimicrobial activity	
H_37_Rv	Standard strain, susceptible to isoniazid and rifampicin	
FJ05189	Clinically isolated multi-drug-resistant strain, resistant to isoniazid and rifampicin	
FJ05120	Clinically isolated multi-drug-resistant strain, resistant to isoniazid and rifampicin	
FJ05195	Clinically isolated extensive-drug-resistant strain, resistant to isoniazid, rifampicin, ethambutol, streptomycin, kanamycin and ofloxacin	
*Plasmids*		
13R-1	Cosmid based on vector pOJ446, containing the majority of sansanmycin biosynthetic gene cluster *ssaM–ssaV* including *ssaX*	[11]
13R-1-SCP2KO	Cosmid 13R-1with the minimal replicon of SCP2* replaced by ampicillin resistance marker *bla*, Amp^r,^ Am^r^	This study
13R-1-SCP2KO-XKO	Cosmid 13R-1-SCP2KO with the in-frame deletion of *ssaX*, Amp^r^, Am^r^	This study
pIJ779	Vector used as the template for amplifying *aadA* cassette, Spec^r^	[21]
pSET152	*Streptomyces* integrative vector, Am^r^	[22]
pL646	pSET152 derivative containing the constitutive promoter ermE*p, Am^r^	[36]
pL-ssaX	pL646 derivative plasmid containing 843 bp complete coding region of *ssaX*	This study

*Amp* ^*r*^ ampicillin resistance, *Am* ^*r*^ apramycin resistance, *Km* kanamycin resistance, *Cm* ^*r*^ chloramphenicol resistance, *Spec* ^*r*^ spectinomycin resistance

### Construction and complementation of *Streptomyces* sp. SS *ssaX* mutant

The *ssaX* in-frame deletion mutant SS/XKO was constructed by the λ-RED mediated PCR targeting method [21], using cosmid 13R-1 [11] covering *ssaM–ssaV* of sansanmycin biosynthetic gene cluster. In order to disrupt *ssaX* through homologous recombination, the minimal replicon of SCP2* of 13R-1 was firstly replaced by ampicillin resistance marker *bla*, resulting 13R-1-SCP2KO. Then, a streptomycin resistance cassette (*aadA* gene) was amplified with primers PT-X-1 (5′-GCGGGAGGCCCCGCTGAACAGGGCCGCGATGCTGTCGTCATTCCGGGGATCCGTCGACC-3′) and PT-X-2 (5′-GTCACCGACACCGCCTATGAGAAGCGCCGCGAGGAGATCTGTAGGCTGGAGCTGCTTC-3′) including two 39-nt homologous extensions to sequences up- and downstream of the target *ssaX* gene. The cassette was introduced into *E. coli* BW25113/pIJ790 to substitute *ssaX* on cosmid 13R-1-SCP2KO. The streptomycin resistance cassette on the correct recombinant cosmid was removed by FLP-recombinase in *E. coli* DH5α/BT340. The mutant cosmid 13R-1-SCP2KO-XKO was introduced into *E. coli* ET12567/pUZ8002 and then transferred into *Streptomyces* sp. SS by conjugation. Double-crossover exconjugants (Am^s^) were selected on MS agar with and without Am and confirmed by PCR using primers PT-X-7 (5′-TGAAGCCCGCCGCCTTTC-3′) and PT-X-8 (5′-TCTGCCTTCCGCCTGACCAT-3′) and southern blot hybridization using DIG Prime DNA Labeling and Detection Starter Kit I (for color detection with NBT/BCIP, Roche). The genomic DNAs were digested with *Bam*HI and hybridized with specific probes of *ssaX* deleted fragment amplified with primers SB-X-1 (5′-CTCGACCTCGTTCATGGAGT-3′) and SB-X-2 (5′-AGTACGTCGACTGGGAGCAC-3′) and the fragment downstream of *ssaX* amplified with primers SB-X-3 (5′-AGAAACCACGATGCGAAATC-3′) and SB-X-4 (5′-TGGATTTTTCGCTTCAAACC-3′) respectively. The resulted *ssaX* deletion mutant was designated SS/XKO.

For complementation analysis, complete *ssaX* coding region was amplified using primers pL-ssaX-F (5′-CGCATATGCAAGGGCATCGCGAC-3′) and pL-ssaX-R (5′-ATAGGATCCTCAGCGCCGGGTGCC-3′), and then cloned into the *Nde*I and *Bam*HI sites of a pSET152-derived expression plasmid, pL646 [23], under the control of a strong constitutive promoter ermE^*^p. The resulted expression vector pL-ssaX was transferred into SS/XKO and *Streptomyces* sp. SS by conjugation to give the complementation strain and *ssaX* overexpression strain respectively. The plasmid pSET152 [22] was transferred to SS/XKO and the wild type strain respectively as controls.

### Analysis of sansanmycin production

Fermentation, isolation, and high-pressure liquid chromatography (HPLC) analysis of sansanmycins were carried out as described previously [1, 25]. In brief, pieces of well-grown agar cultures of different strains were firstly inoculated in fermentation medium and cultured at 28 °C for 48 h at 200 rpm. The obtained seed cultures were trans-inoculated into three parallel 100 ml fermentation medium by 5 % inoculation and grown at 28 °C for 5 days at 200 rpm. In the feeding test, each exogenous substrate was added to the fermentation medium to the final concentration of 3 mM. At indicated time points, five-milliliter cell cultures were collected by centrifugation and dried at 60 °C to constant weight for monitoring the growth curve. The obtained supernatants were analyzed for antibacterial activity and production of sansanmycins by bioassay and HPLC. Antibacterial activity was measured by cylinder plate method using *P. aeruginosa* 11. For analyzing the expected analogues, the supernatant of fermentation broth was enriched by Sep-Pak C_18_ Classic Cartridge (Waters Associates, Milford, MA, USA), eluted with 60 % methanol solution. The effluent was subjected to HPLC on an XBridge™ C_18_ column (4.6 × 150 mm, 3.5 μm, Waters, Dublin, Ireland) maintained at 40 °C, with a gradient of 80:20 0.1 % (w/v) (NH_4_)_2_CO_3_-MeOH to 40:60 in 40 min as mobile phase at a flow rate of 1 ml/min. Absorbance was monitored at 254 nm. For the analysis of sansanmycin MX-3 and MX-6, the mobile phase was changed to 10:90 MeOH-H_2_O (pH adjusted to 12.0 with NH_3_·H_2_O) in 40 min.

### Purification of sansanmycin analogues

Isolation and purification of sansanmycin analogues was performed following the method of Xie et al. [25] with some modifications. Fifty liters of fermentation supernatant was obtained by centrifugation and then applied on a column of macroporous absorbant resin 4006. The active materials were eluted with 30 % aqueous acetone. Then the effluent was applied on Toyopearl DEAE-Sephadex A25 eluted with Tris-HCl (20 mM, pH 8.5) plus NaCl and monitored by HPLC-UV. The concentration of NaCl was adjusted with different compounds from 0.01 to 0.05 M. The effluent containing target compounds was collected and further purified by preparative HPLC (YMC-Pack ODS-A 5 μm, 250 × 20 mm column, 0.1 % (w/v) (NH_4_)_2_CO_3_-MeOH; flow rate, 5 ml/min; UV detection at 254 nm and oven temperature at 40 °C). The ratio of 0.1 % (w/v) (NH_4_)_2_CO_3_ and MeOH was dependent on different compounds. The structures of obtained compounds were determined using ESI-MS and ESI-MS/MS (ThermoFisher LTQ Orbitrap XL mass spectrometer) as well as NMR [Varian Mercury 600 spectrometers, in dimethyl sulfoxide (DMSO)-*d*_*6*_].

### Antibacterial assay

The minimum inhibitory concentrations (MICs) for *M. tuberculosis* strains were determined by the microplate Alamar blue assay (MABA) [41]. All *M. tuberculosis* strains were grown on Middlebrook 7H9 medium supplemented with 0.2 % (v/v) glycerol and 10 % (v/v) OADC (oleic acid, albumin, dextrose, catalase) until the mid-log phase of growth at 37 °C. The final suspension of bacteria cells were diluted in Middlebrook 7H9 medium to 10^6^ cfu/ml. Initial compound dilutions were prepared in DMSO, and subsequent twofold dilutions were performed in 100 μl of 7H9 (no Tween 80) in the microplates. Then, the MIC was measured in sterile 96-well plates with 100 μl of the bacterial suspension and 100 μl compound dilution per well. The MIC was defined as the lowest concentration of drug that prevented the color change of Alamar blue reagent from blue to pink. Rifampicin, isoniazid, ethambutol and streptomycin were used as controls.

The MICs for other bacterial strains were determined by a microdilution test following recommendations from the Clinical and Laboratory Standards Institute (CLSI, formerly NCCLS) [42]. The bacterial strains were grown on Mueller–Hinton broth (MHB) [43], and the final suspension of bacteria (in MHB medium) was adjusted to 10^6^ cells/ml. The dilutions of tested compounds were performed as method above with MHB medium instead. Then serial dilutions (100 μl) were transferred to a 96-well plate in triplicate, and 100 μl of the bacterial suspension was added to each well. After incubation at 37 °C for 24 h, the MIC was defined as the lowest concentration that inhibited the growth of the tested organism detected by visual observation. Streptomycin was used as the positive control.

### Stability determination of sansanmycin analogues

To dissect the stability of sansanmycin A and other sansanmycin analogues, compounds were dissolved in 0.05 M KH_2_PO_4_ buffer (pH adjusted to 6.0 with NaOH). All samples were incubated at 25 °C for 9 days. Each sample has three parallel repeats. Residual analogues were analyzed by HPLC and quantified by the peak areas.

## Additional file

10.1186/s12934-016-0471-1 **Table S1**. ^1^H NMR (600 MHz) and ^13^C NMR (150 MHz) Data for sansanmycin MX-1. **Table S2**. ^1^H NMR (600 MHz) and ^13^C NMR (150 MHz) Data for sansanmycin MX-2. **Table S3**. ^1^H NMR (600 MHz) and ^13^C NMR (150 MHz) Data for sansanmycin MX-4. **Table S4**. ^1^H NMR (600 MHz) and ^13^C NMR (150 MHz) Data for sansanmycin MX-6. **Figure S1**. Selected 2D NMR correlations for sansanmycin MX-2. **Figure S2**. Selected 2D NMR correlations for sansanmycin MX-4. **Figure S3**. Selected 2D NMR correlations for sansanmycin MX-6. **Figure S4**. ^1^H NMR spectrum of sansanmycin MX-2 (600 MHz, DMSO-*d*_*6*_). **Figure S5**. ^13^C NMR spectrum of sansanmycin MX-2 (150 MHz, DMSO-*d*_*6*_). **Figure S6**. ^1^H NMR spectrum of sansanmycin MX-4 (600 MHz, D_2_O, pD = 8.5). **Figure S7**. ^13^C NMR spectrum of sansanmycin MX-4 (150 MHz, D_2_O, pD = 8.5). **Figure S8**. ^1^H NMR spectrum of sansanmycin MX-6 (600 MHz, DMSO-*d*_*6*_). **Figure S9**. ^13^C NMR spectrum of sansanmycin MX-6 (150 MHz, DMSO-*d*_*6*_). **Figure S10**. ^1^H NMR spectrum of sansanmycin MX-3 (600 MHz, DMSO-*d*_*6*_). **Figure S11**. ^1^H NMR spectrum of sansanmycin MX-5 (600 MHz, DMSO-*d*_*6*_).
