# Supplementary material for: Improving the N-terminal diversity of sansanmycin through mutasynthesis
Source: Microb Cell Fact. 2016 May 6;15:77. doi: 10.1186/s12934-016-0471-1 (PMC4858918; doi:10.1186/s12934-016-0471-1)

**Supplementary Materials**

**Improving the N-terminal diversity of sansanmycin through mutasynthesis**

Yuanyuan Shi, Zhibo Jiang, Xuan Lei, Ningning Zhang, Qiang Cai, Qinglian Li, Lifei Wang, Shuyi Si, Yunying Xie^*^, Bin Hong^*^

The Key Laboratory of Biotechnology of Antibiotics of Ministry of Health, Institute of Medicinal Biotechnology, Chinese Academy of Medical Sciences & Peking Union Medical College, No.1 Tiantan Xili, Beijing 100050, China

*To whom correspondence should be addressed.

Email: [binhong69@hotmail.com](mailto:binhong69@hotmail.com); [hongbin@imb.pumc.edu.cn](mailto:hongbin@imb.pumc.edu.cn) (Bin Hong); [xieyy@imb.pumc.edu.cn](mailto:xieyy@imb.pumc.edu.cn) (Yunying Xie).

**Table of Contents**

Supplementary Tables and Figures 2

Table S1. ^1^H NMR (600 MHz) and ^13^C NMR (150 MHz) Data for sansanmycin MX-1 2

Table S2. ^1^H NMR (600 MHz) and ^13^C NMR (150 MHz) Data for sansanmycin MX-2 3

Table S3. ^1^H NMR (600 MHz) and ^13^C NMR (150 MHz) Data for sansanmycin MX-4 4

Table S4. ^1^H NMR (600 MHz) and ^13^C NMR (150MHz) Data for sansanmycin MX-6 5

Figure S1. Selected 2D NMR correlations for sansanmycin MX-2 6

Figure S2. Selected 2D NMR correlations for sansanmycin MX-4 7

Figure S3. Selected 2D NMR correlations for sansanmycin MX-6 8

Figure S4. ^1^H NMR spectrum of sansanmycin MX-2 (600 MHz, DMSO-*d_6_*) 9

Figure S5. ^13^C NMR spectrum of sansanmycin MX-2 (150 MHz, DMSO-*d_6_*) 10

Figure S6. ^1^H NMR spectrum of sansanmycin MX-4 (600 MHz, D_2_O, pD = 8.5) 11

Figure S7. ^13^C NMR spectrum of sansanmycin MX-4 (150 MHz, D_2_O, pD = 8.5) 12

Figure S8. ^1^H NMR spectrum of sansanmycin MX-6 (600 MHz, DMSO-*d_6_*) 13

Figure S9. ^13^C NMR spectrum of sansanmycin MX-6 (150 MHz, DMSO-*d_6_*) 14

Figure S10. ^1^H NMR spectrum of sansanmycin MX-3 (600 MHz, DMSO-*d_6_*) 15

Figure S11. ^1^H NMR spectrum of sansanmycin MX-5 (600 MHz, DMSO-*d_6_*) 16

# Supplementary Tables and Figures

## Table S1. ^1^H NMR (600 MHz) and ^13^C NMR (150 MHz) Data for sansanmycin MX-1

| **Position*** | **Multiplicity** | **δ_C_** | **δ_H_ (*J*, Hz)** | **Extra signals due to conformers** | |
| --- | --- | --- | --- | --- | --- |
|  |  |  |  | **δ_C_** | **δ_H_ (*J*, Hz)** |
| uracil-2 | N-CO-N | 150.6 |  |  |  |
| uracil-4 | CO-N | 163.3 |  |  |  |
| uracil-5 | CH | 102.2 | 5.63, m |  |  |
| uracil-6 | CH | 140.3 | 7.46, m |  |  |
| sugar-1 | O-CH-N | 92.2 | 5.97, d (3.3) |  | 8.51, d (9.2) |
| sugar-2 | O-CH | 71.6 | 4.41, m | 69.9 | 3.49, s |
| sugar-3 | CH_2_ | 34.0 | 2.52, d (3.7) | 36.2 | 2.27, m |
|  |  |  | 2.88, dd (17.1,6.3) |  |  |
| sugar-4 | >C= | 140.4 |  |  |  |
| sugar-5 | -CH= | 96.9 | 5.90, d (9.4) |  | 9.57, d (9.4)  9.76, m |
| DABA-1 | CO-N | 167.6 |  |  |  |
| DABA-2 | CH | 57.2 | 4.60, m |  |  |
| DABA-3 | CH | 54.4 | 4.50, m | 55.8 |  |
| DABA-4 | CH_3_ | 15.9 | 0.88,d (6.5) |  | 1.22, s |
| DABA-N-CH_3_ | N-CH_3_ | 32.9 | 2.74, m | 33.3 | 2.17, s |
| Trp-1 | -COOH | 175.2 |  |  |  |
| Trp-2 | CH | 52.5 | 3.34, m |  | 4.98, m |
| Trp-3 | CH_2_ | 32.2 | 3.58, d (9.0) |  | 3.06, m |
|  |  |  | 3.21, m |  | 3.45, d (8.2) |
| Trp-2′ | CH | 123.4 | 7.12, d (7.6) |  |  |
| Trp-3′ | ArC | 111.7 |  |  |  |
| Trp-3a′ | ArC | 128.3 |  |  |  |
| Trp-4′ | ArCH | 118.9 | 7.52, d (5.6) |  | 7.77, s |
| Trp-5′ | ArCH | 117.7 | 6.85, m |  |  |
| Trp-6′ | ArCH | 120.1 | 6.94, t (7.5) |  |  |
| Trp-7′ | ArCH | 110.8 | 7.22, d (8.0) | 110.7 | 7.05, s |
| Trp-7a′ | ArC | 135.8 |  |  |  |
| ureido | N-CO-N | 157.4 |  |  |  |
| Met-1 | CO-N | 172.6 |  |  |  |
| Met-2 | CH | 50.5 | 4.15, m |  |  |
| Met-3 | CH_2_ | 32.1 | 1.80, m | 33.0 | 1.68, m |
| Met-4 | CH_2_ | 29.6 | 2.42, m |  | 2.39, m |
| Met-S-CH_3_ | CH_3_ | 14.6 | 1.99, s |  |  |

The spectra were recorded in DMSO. The chemical shifts (δ) are given in ppm.

*Abbreviation for the structure units are: Trp = tryptophan, DABA = 2-amino-3-methyl-aminobutyric acid, Met = Methionine

## Table S2. ^1^H NMR (600 MHz) and ^13^C NMR (150 MHz) Data for sansanmycin MX-2

| **Position*** | **Multiplicity** | **δ_C_** | **δ_H_ (*J*, Hz)** | **Extra signals due to conformers** | |
| --- | --- | --- | --- | --- | --- |
|  |  |  |  | **δ_C_** | **δ_H_ (*J*, Hz)** |
| uracil-2 | N-CO-N | 174.5 |  |  |  |
| uracil-4 | CO-N | 157.3 |  |  |  |
| uracil-5 | CH | 102.2 | 5.67, d (8.1) |  | 5.47, d (8.0) |
| uracil-6 | CH | 140.3 | 7.45, d (8.2) |  | 7.11, d (8.1) |
| sugar-1 | O-CH-N | 91.8 | 5.96, t (3.6) | 92.1 |  |
| sugar-2 | O-CH | 71.6 | 4.40, m | 71.5 | 4.27, m |
| sugar-3 | CH_2_ | 34.0 | 2.84, d (6.4) | 33.9 | 2.47, d (5.1) |
|  |  |  | 2.78, d (6.2) |  |  |
| sugar-4 | >C= | 141.3 |  | 140.5 |  |
| sugar-5 | -CH= | 96.9 | 5.82, m | 102.2 | 5.67, m |
| DABA-1 | CO-N | 166.9 |  | 166.6 |  |
| DABA-2 | CH | 55.1 | 4.60, m |  |  |
| DABA-3 | CH | 53.5 | 4.85, m |  |  |
| DABA-4 | CH_3_ | 14.0 | 1.03, d (6.8) | 14.6 | 0.62, d (6.3) |
| DABA-N-CH_3_ | N-CH_3_ | 27.2 | 2.55, s |  |  |
| Tyr-1 | CO-N | 175.2 |  |  |  |
| Tyr -2 | CH | 54.9 | 4.15, m |  |  |
| Tyr -3 | CH_2_ | 28.2 | 3.08, m |  |  |
|  |  |  | 2.97, dd (14.0,5.4) |  |  |
| Tyr -1′ | ArC | 135.9 |  |  |  |
| Tyr -2′ | ArCH | 120.4 | 6.97, d (2.3) | 118.0 | 6.90, m |
| Tyr -3′ | ArCH | 118.7 | 7.51, d(7.9) |  | 7.27, m |
| Tyr -4′ | ArC-O | 155.7 |  |  |  |
| Tyr -5′ | ArCH | 118.7 | 7.51, d(7.9) |  | 7.27, m |
| Tyr -6′ | ArCH | 120.4 | 6.97, d (2.3) | 118.0 | 6.90, m |
| Trp-1 | -COOH | 175.0 |  |  |  |
| Trp-2 | CH | 52.6 | 3.65,dd (8.7,3.8) |  |  |
| Trp-3 | CH_2_ | 39.8 | 2.69, m  2.21, m |  |  |
| Trp-2′ | CH | 130.0 | 6.90, m  6.97, m | 130.1 |  |
| Trp-3′ | ArC | 111.0 |  |  |  |
| Trp-3a′ | ArC | 127.8 |  | 128.0 |  |
| Trp-4′ | ArCH | 124.9 | 7.51, d (7.9) |  |  |
| Trp-5′ | ArCH | 123.4 | 7.07, m |  |  |
| Trp-6′ | ArCH | 115.0 | 6.65, t (8.5) | 114.9 |  |
| Trp-7′ | ArCH | 110.9 | 7.27, m |  |  |
| Trp-7a′ | ArC | 128.5 |  |  |  |
| ureido | N-CO-N | 162.9 |  |  |  |
| Met-1 | CO-N | 172.0 |  |  |  |
| Met-2 | CH | 51.6 | 4.07, m  4.01, m |  |  |
| Met-3 | CH_2_ | 32.3 | 1.77, m | 32.2 |  |
|  |  |  | 1.67, m |  |  |
| Met-4 | CH_2_ | 29.6 | 2.36, m  2.87, s |  |  |
| Met-S-CH_3_ | CH_3_ | 14.0 | 1.97, s |  |  |

The spectra were recorded in DMSO. The chemical shifts (δ) are given in ppm.

*Abbreviation for the structure units are: Tyr = tyrosine, Trp = tryptophan, DABA = 2-amino-3-methyl-aminobutyric acid, Met = Methionine

## Table S3. ^1^H NMR (600 MHz) and ^13^C NMR (150 MHz) Data for sansanmycin MX-4

| **Position*** | **Multiplicity** | **δ_C_** | **δ_H_ (*J*, Hz)** | **Extra signals due to conformers** | |
| --- | --- | --- | --- | --- | --- |
|  |  |  |  | **δ_C_** | **δ_H_ (*J*, Hz)** |
| Uracil-2 | N-CO-N | 161.3 |  |  |  |
| Uracil-4 | CO-N | 179.4 |  |  |  |
| Uracil-5 | CH | 105.6 | 5.51, d (9.0) | 105.6 | 5.81, d (9.0) |
| Uracil-6 | CH | 141.2 | 6.88, d (9.0) | 141. 8 | 7.21, d (8.6) |
| Sugar-1 | O-CH-N | 96.2 | 6.07, s | 96.6 | 6.09, s |
| Sugar-2 | O-CH | 75.9 | 4.47, m | 75.5 | 4.33, m |
| Sugar-3 | CH_2_ | 35.8 | 2.75, m | 35.6 | 2.56, m |
|  |  |  | 2.78, m |  | 2.62, m |
| Sugar-4 | >C= | 147.5 |  | 147.2 |  |
| Sugar-5 | -CH= | 99.3 | 5.90, s | 98.9 | 5.94, s |
| DABA-1 | CO-N | 170.2 |  | 170.0 |  |
| DABA-2 | CH | 58.5 | 4.55, m | 58.9 | 4.44, m |
| DABA-3 | CH | 55.9 | 4.54, m |  |  |
| DABA-4 | CH_3_ | 15.9 | 1.12, d (7.0) | 16.5 | 0.46, d (7.0) |
| DABA-N-CH_3_ | N-CH_3_ | 32.9 | 2.85, s |  |  |
| Phe-1 | CO-N | 179.9 |  | 179.4 |  |
| Phe-2 | CH | 55.8 | 3.92, m | 56.1 | 4.20, m |
| Phe-3 | CH_2_ | 43.8 | 2.73, m | 42.8 | 2.58, m |
|  |  |  | 2.86, m |  | 2.80, m |
| Phe-1′ | ArC | 139.7 |  | 139.5 |  |
| Phe-2′ | ArCH | 132.1 | 7.15, m |  |  |
| Phe-3′ | ArCH | 131.5 | 7.34, m | 131.7 |  |
| Phe-4′ | ArCH | 129.8 | 7.27, m |  |  |
| Phe-5′ | ArCH | 131.5 | 7.34, m | 131.7 |  |
| Phe-6′ | ArCH | 132.1 | 7.15, m |  |  |
| Trp-1 | -COOH | 182.2 |  |  |  |
| Trp-2 | CH | 59.1 | 4.37, m |  |  |
| Trp-3 | CH_2_ | 31.2 | 3.28, m |  |  |
|  |  |  | 3.08, m |  |  |
| Trp-2′ | CH | 127.0 | 7.21, m |  |  |
| Trp-3′ | ArC | 113.4 |  | 113.4 |  |
| Trp-3a′ | ArC | 130.2 |  |  |  |
| Trp-4′ | ArCH | 121.6 | 7.68, m |  |  |
| Trp-5′ | ArCH | 121.9 | 7.17, m |  |  |
| Trp-6′ | ArCH | 124.5 | 7.23, m |  |  |
| Trp-7′ | ArCH | 114.5 | 7.48, m |  |  |
| Trp-7a′ | ArC | 138.9 |  |  |  |
| Ureido | N-CO-N | 161.3 |  | 161.2 |  |
| Met-1 | CO-N | 177.3 |  | 177.3 |  |
| Met-2 | CH | 54.6 | 4.23, m | 54.5 | 4.22, m |
| Met-3 | CH_2_ | 33.5 | 1.90, m |  |  |
|  |  |  | 1.79, m |  |  |
| Met-4 | CH_2_ | 32.1 | 2.41, m |  |  |
| Met-S-CH_3_ | CH_3_ | 17.0 | 2.01, s |  |  |

The spectra were recorded in D_2_O, pD = 8.5. The chemical shifts (δ) are given in ppm.*Abbreviation for the structure units are: *m*-Tyr = *meta*-tyrosine, Phe = phenylalanine, DABA = 2-amino-3-methyl-aminobutyric acid, Met = methionine.

## Table S4. ^1^H NMR (600 MHz) and ^13^C NMR (150MHz) Data for sansanmycin MX-6

| **Position*** | **Multiplicity** | | **δ_C_** | **δ_H_ (*J*, Hz)** | **Extra signals due to conformers** | |
| --- | --- | --- | --- | --- | --- | --- |
|  |  |  |  |  | **δ_C_** | **δ_H_ (*J*, Hz)** |
| uracil-2 | N-CO-N | 152.0 | |  |  |  |
| uracil-4 | CO-N | 172.0 | |  |  |  |
| uracil-5 | CH | 102.3 | | 5.63, m |  |  |
| uracil-6 | CH | 140.1 | | 7.39, m |  |  |
| sugar-1 | O-CH-N | 92.4 | | 5.99, d (10.7) |  |  |
| sugar-2 | O-CH | 72.5 | | 4.38, s | 70.3 | 3.50,s |
| sugar-3 | CH_2_ | 34.3 | | 2.87, d (10.1)  2.47, m |  |  |
| sugar-4 | >C= | 157.7 | |  |  |  |
| sugar-5 | -CH= | 96.7 | | 5.78, d (9.2) |  |  |
| DABA-1 | CO-N | 166.6 | |  |  |  |
| DABA-2 | CH | 55.2 | | 4.58, m  4.73, m |  |  |
| DABA-3 | CH | 49.8 | | 4.89, m |  |  |
| DABA-4 | CH_3_ | 14.6 | | 1.01, d (6.8) | 15.3 | 1.13, d (6.4) |
| DABA-N-CH_3_ | N-CH_3_ | 34.2 | | 2.83, s | 34.3 |  |
| ureido | N-CO-N | 167.2 | |  |  |  |
| Trp-1 | -COOH | 177.4 | |  |  |  |
| Trp-2 | CH | 53.2 | | 4.31, m |  | 4.01, m |
| Trp-3 | CH_2_ | 32.7 | | 3.88, s |  | 2.96, d (9.6) |
| Trp-2′ | ArCH | 123.3 | | 6.97, t (7.3) | 117.8 | 6.89, t (7.3) |
| Trp-3′ | ArC | 112.3 | |  |  |  |
| Trp-3a′ | ArC | 136.4 | |  |  |  |
| Trp-4′ | ArCH | 128.3 | | 7.81, d (8.2) |  | 7.91, d (8.4) |
| Trp-5′ | ArCH | 124.5 | | 7.07, m | 127.9 |  |
| Trp-6′ | ArCH | 111.9 | | 7.25, m |  |  |
| Trp-7′ | ArCH | 125.2 | | 7.52, d (7.8) | 126.5 | 7.71, d (8.3) |
| Trp-7a′ | ArC | 135.9 | |  |  |  |
| Met-1  Met-2 | CO-N  CH | 175.4  53.6 | | 4.13, d (5.5) |  |  |
| Met-3 | CH_2_ | 34.1 | | 1.68, m |  |  |
| Met-4 | CH_2_ | 29.9 | | 2.37, m  2.47, m |  |  |
| Met-S-CH_3_ | CH_3_ | 14.0 | | 1.99, s |  |  |
| Nt-Met-1 | CO-N | 175.7 | |  |  |  |
| Nt-Met-2 | CH | 53.2 | | 4.31, s |  |  |
| Nt-Met-3 | CH_2_ | 34.3 | | 1.81, m |  |  |
| Nt-Met-4 | CH_2_ | 29.9 | | 2.47, m |  |  |
|  |  |  | | 2.37, m |  |  |
| Nt-Met-S-CH_3_ | CH_3_ | 15.3 | | 1.99, s |  |  |

The spectra were recorded in slightly alkaline DMSO. The chemical shifts (δ) are given in ppm.*Abbreviation for the structure units are: Trp = tryptophan, Met = methionine, DABA = 2-amino-3-methyl-aminobutyric acid, Trp = tryptophan, Nt-Met = N-terminal methionine.

## Figure S1. Selected 2D NMR correlations for sansanmycin MX-2

## Figure S2. Selected 2D NMR correlations for sansanmycin MX-4

## Figure S3. Selected 2D NMR correlations for sansanmycin MX-6

## Figure S4. ^1^H NMR spectrum of sansanmycin MX-2 (600 MHz, DMSO-*d_6_*)


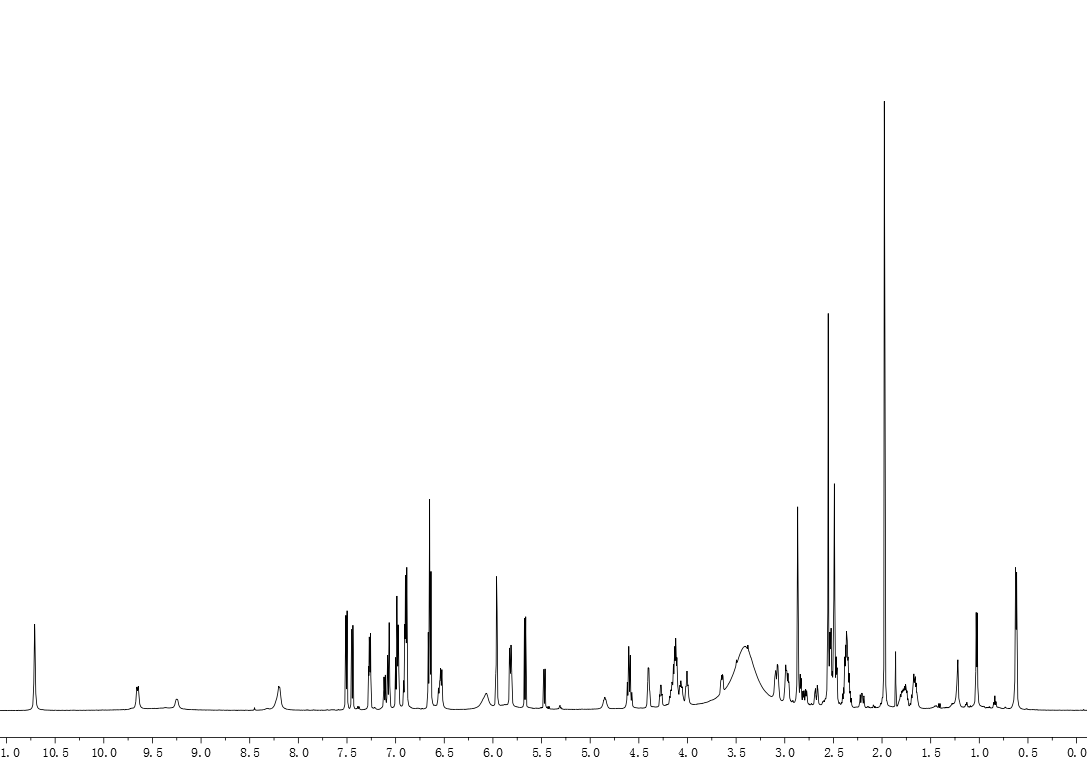


## Figure S5. ^13^C NMR spectrum of sansanmycin MX-2 (150 MHz, DMSO-*d_6_*)


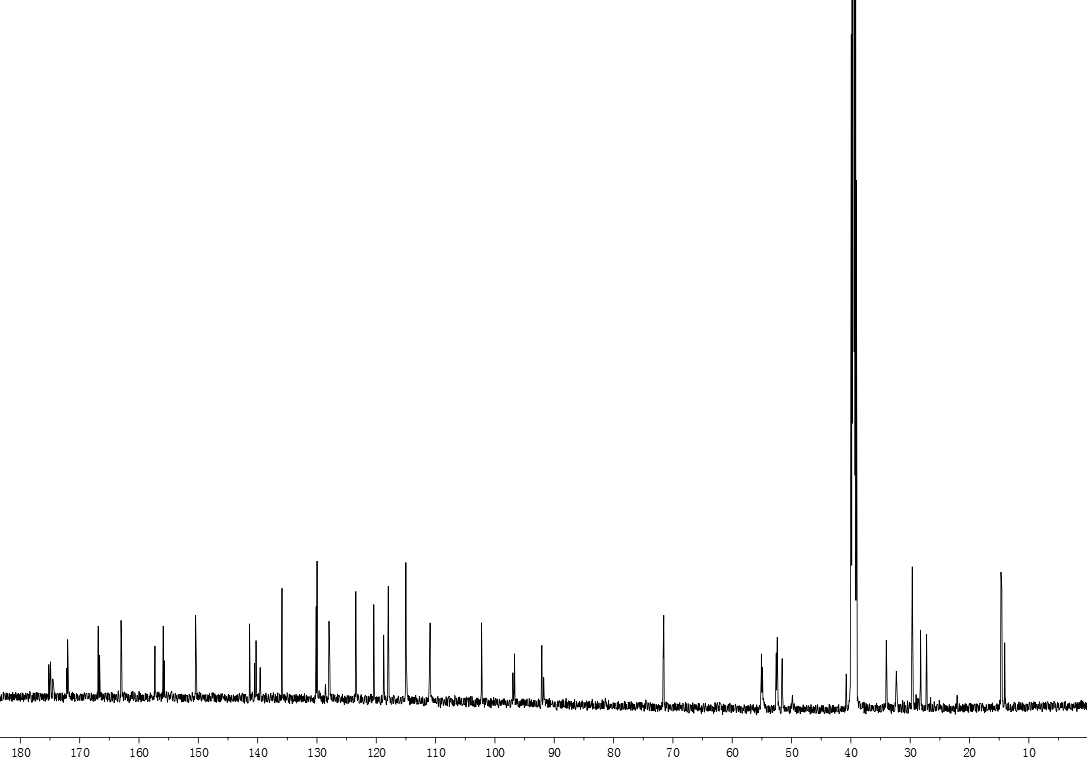


## Figure S6. ^1^H NMR spectrum of sansanmycin MX-4 (600 MHz, D_2_O, pD = 8.5)


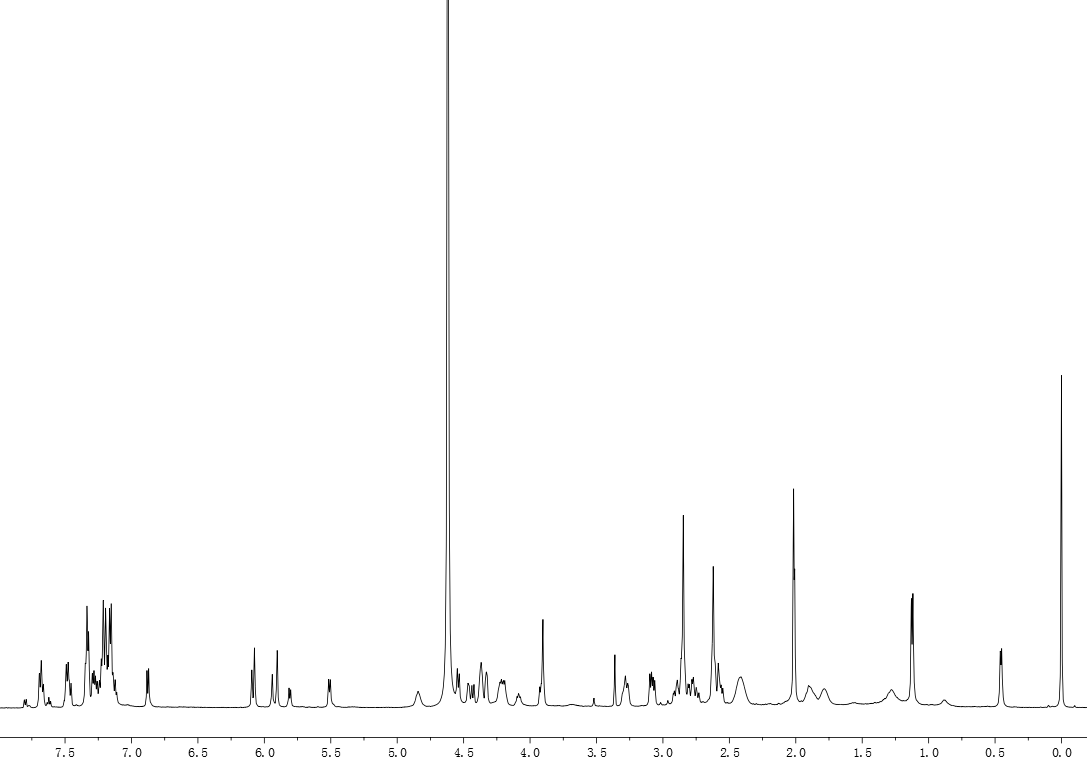


## Figure S7. ^13^C NMR spectrum of sansanmycin MX-4 (150 MHz, D_2_O, pD = 8.5)


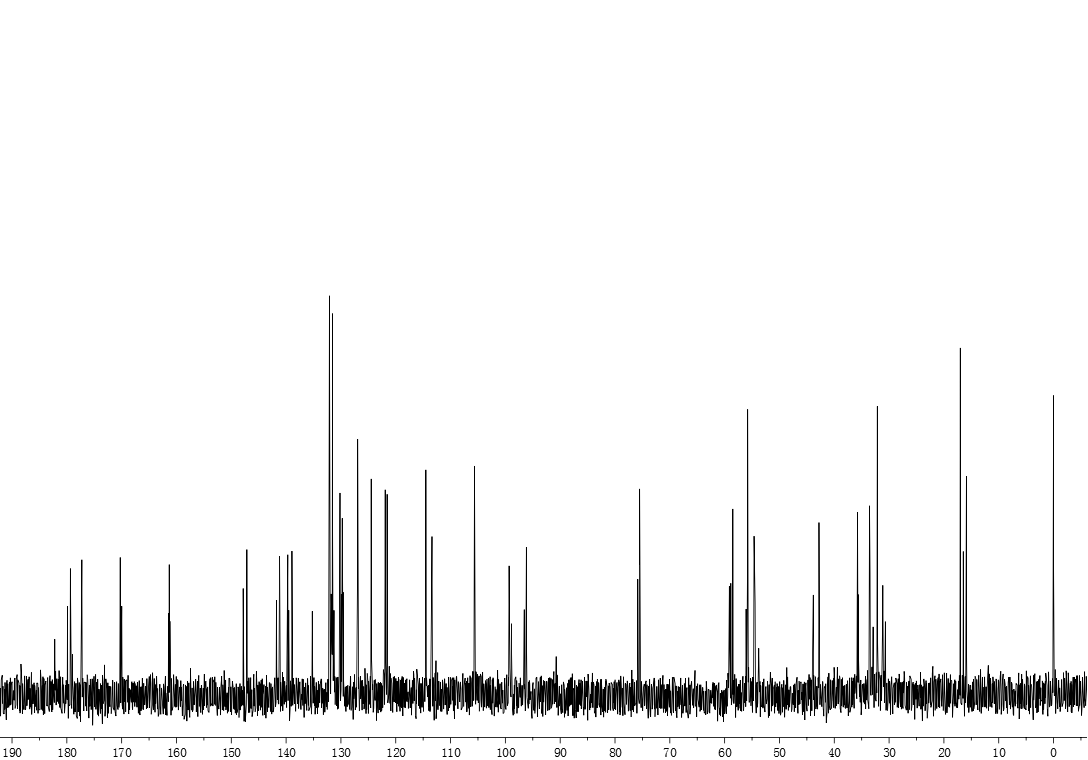


## Figure S8. ^1^H NMR spectrum of sansanmycin MX-6 (600 MHz, DMSO-*d_6_*)


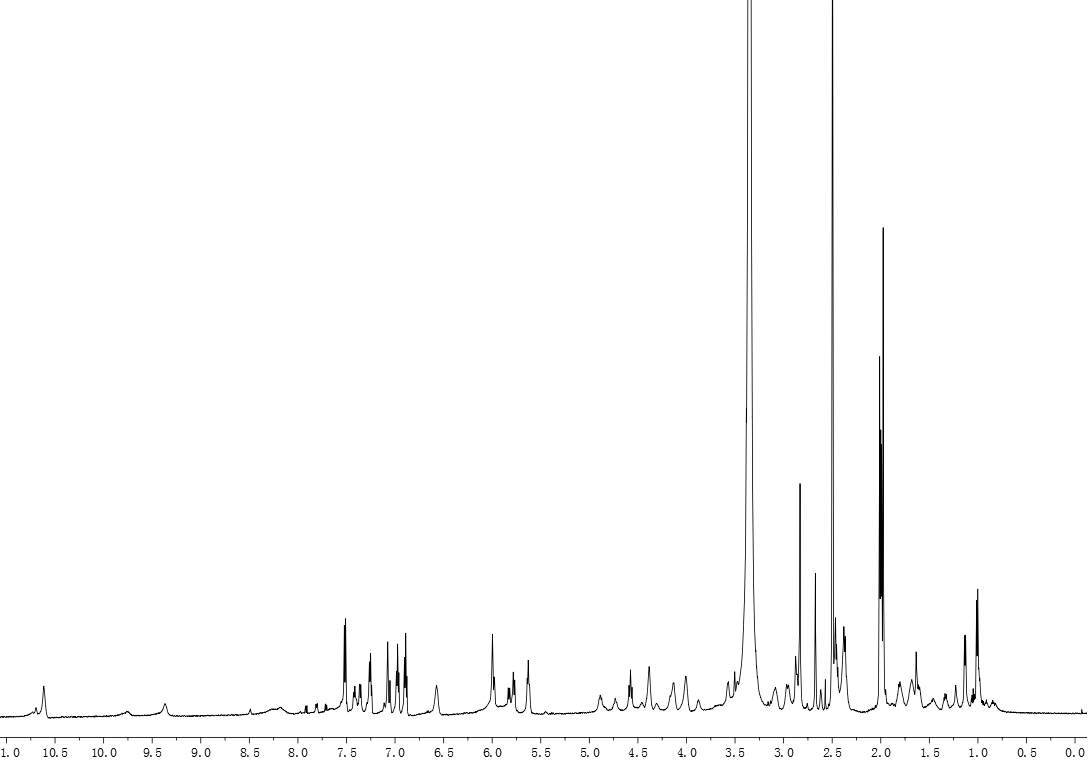


## Figure S9. ^13^C NMR spectrum of sansanmycin MX-6 (150 MHz, DMSO-*d_6_*)


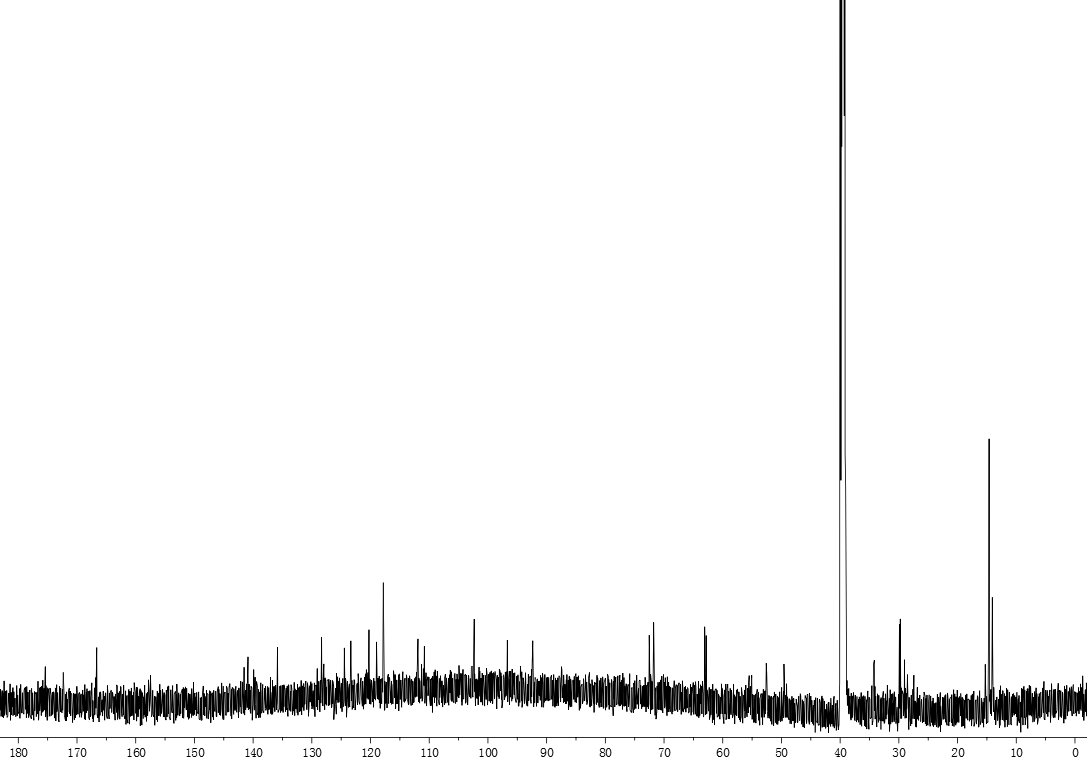


## Figure S10. ^1^H NMR spectrum of sansanmycin MX-3 (600 MHz, DMSO-*d_6_*)


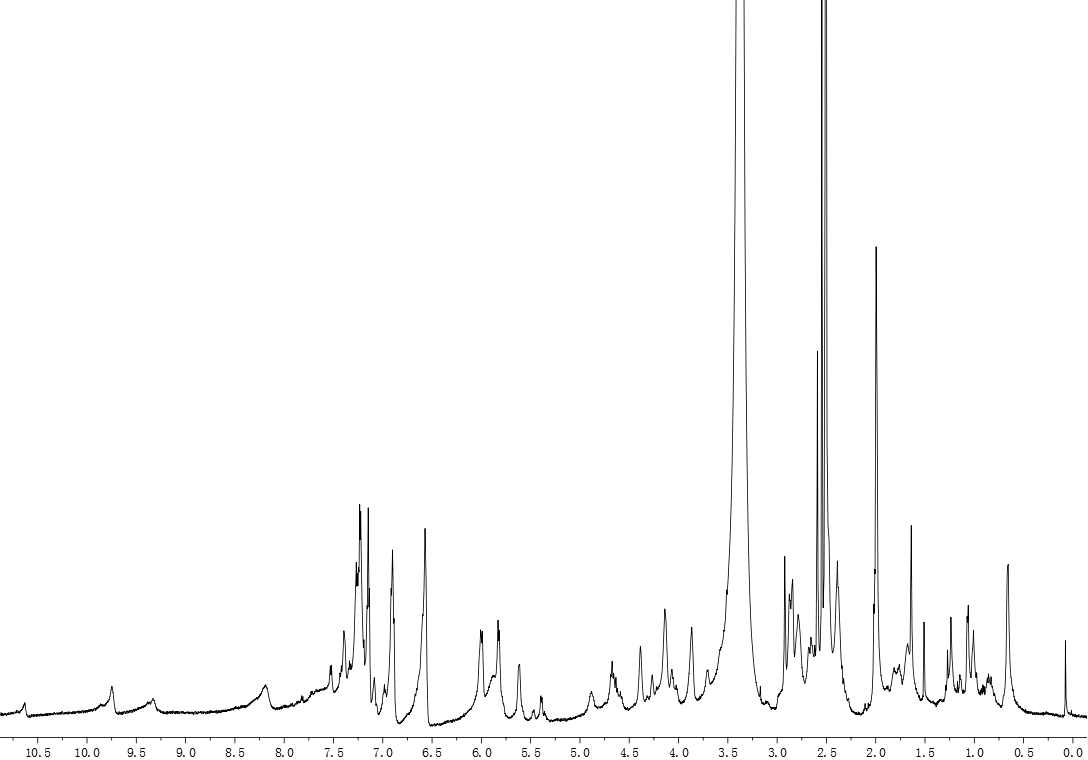

## Figure S11. ^1^H NMR spectrum of sansanmycin MX-5 (600 MHz, DMSO-*d_6_*)


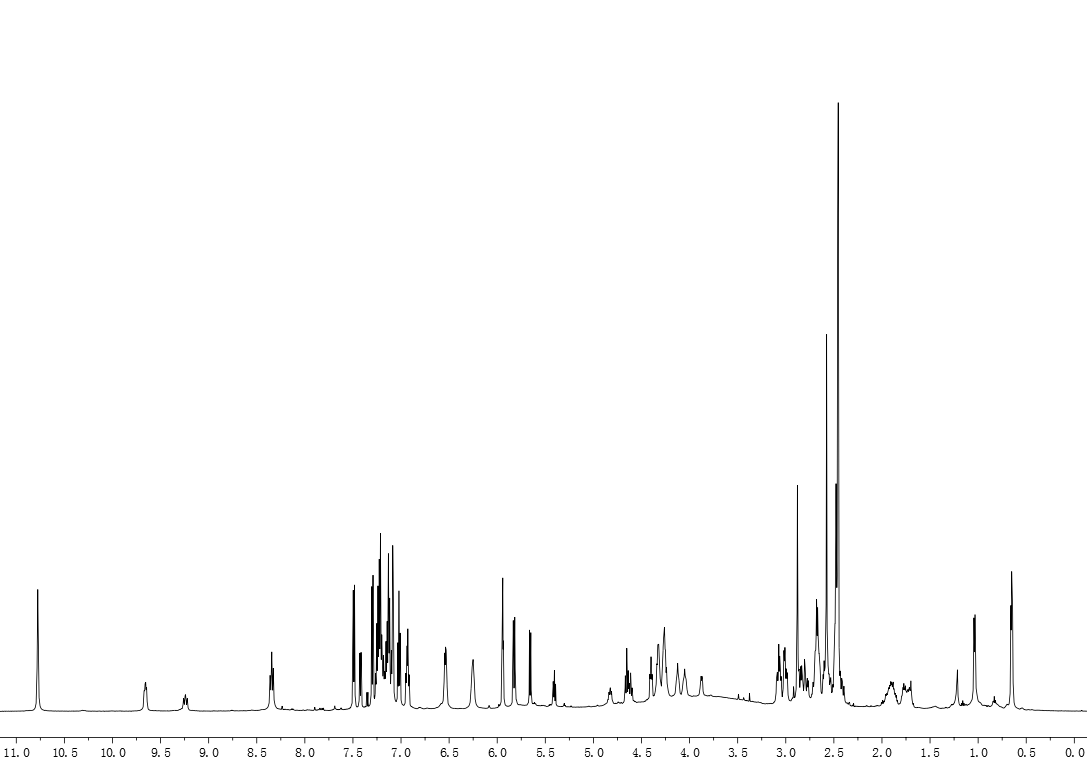

Supplement: Supplementary file 1 — 10.1186/s12934-016-0471-1 Table S1. 1H NMR (600 MHz) and 13C NMR (150 MHz) Data for sansanmycin MX-1. Table S2. 1H NMR (600 MHz) and 13C NMR (150 MHz) Data for sansanmycin MX-2. Table S3. 1H NMR (600 MHz) and 13C NMR (150 MHz) Data for sansanmycin MX-4. Table S4. 1H NMR (600 MHz) and 13C NMR (150 MHz) Data for sansanmycin MX-6. Figure S1. Selected 2D NMR correlations for sansanmycin MX-2. Figure S2. Selected 2D NMR correlations for sansanmycin MX-4. Figure S3. Selected 2D NMR correlations for sansanmycin MX-6. Figure S4. 1H NMR spectrum of sansanmycin MX-2 (600 MHz, DMSO-d 6). Figure S5. 13C NMR spectrum of sansanmycin MX-2 (150 MHz, DMSO-d 6). Figure S6. 1H NMR spectrum of sansanmycin MX-4 (600 MHz, D2O, pD = 8.5). Figure S7. 13C NMR spectrum of sansanmycin MX-4 (150 MHz, D2O, pD = 8.5). Figure S8. 1H NMR spectrum of sansanmycin MX-6 (600 MHz, DMSO-d 6). Figure S9. 13C NMR spectrum of sansanmycin MX-6 (150 MHz, DMSO-d 6). Figure S10. 1H NMR spectrum of sansanmycin MX-3 (600 MHz, DMSO-d 6). Figure S11. 1H NMR spectrum of sansanmycin MX-5 (600 MHz, DMSO-d 6). [file 12934_2016_471_MOESM1_ESM.docx]
